# Dexamethasone Eye Drops to Prevent Treatment-Requiring Retinopathy of Prematurity: The DROPROP Randomized Clinical Trial

**DOI:** 10.1001/jamapediatrics.2026.3247

**Published:** 2026-08-03

**Authors:** Ann Hellström, Mariya Petrishka-Lozenska, Ulrika Sjöbom, Jenny Wallander, Anders K. Nilsson, Chatarina Löfqvist, David Ley, Lotta Gränse, Hanna Maria Öhnell, Anna-Lena Hård, Gunnar Jakobsson, Karin Sävman, Magnus Domellöf, Stefan Löfgren, Eva Larsson, Lois E.H. Smith, Aldina Pivodic, Pia Lundgren

**Affiliations:** 1 Sahlgrenska Centre for Pediatric Ophthalmology Research, Department of Clinical Neuroscience, Institute of Neuroscience and Physiology, Sahlgrenska Academy, University of Gothenburg, Gothenburg, Sweden; 2 Department of Ophthalmology, Sahlgrenska University Hospital, Region Västra Götaland, Gothenburg, Sweden; 3 Learning and Leadership for Health Care Professionals, Institute of Health and Care Science at Sahlgrenska Academy, University of Gothenburg, Gothenburg, Sweden; 4 Department of Ophthalmology, Region Jönköping County, Jönköping, Sweden; 5 Pediatrics, Department of Clinical Sciences, Lund, Lund University, Skåne University Hospital, Lund, Sweden; 6 Ophthalmology, Department of Clinical Sciences, Lund, Lund University, Skåne University Hospital, Lund, Sweden; 7 Department of Pediatrics, Institute of Clinical Sciences, Sahlgrenska Academy, University of Gothenburg, Gothenburg, Sweden; 8 Department of Neonatology, Region Västra Götaland, The Queen Silvia Children’s Hospital, Sahlgrenska University Hospital, Gothenburg, Sweden; 9 Pediatrics, Department of Clinical Sciences, Umeå University, Umeå, Sweden; 10 Department of Paediatric Ophthalmology, Strabismus and Electrophysiology, St Erik Eye Hospital, Stockholm, Sweden; 11 Department of Women’s and Children’s Health, Uppsala University, Uppsala, Sweden; 12 Department of Ophthalmology, Boston Children’s Hospital, Harvard Medical School, Boston, Massachusetts

## Abstract

**Importance:**

Current treatment for type 1 retinopathy of prematurity (ROP), including laser photocoagulation and intravitreal anti–vascular endothelial growth factor therapy, is invasive but necessary to prevent blindness. Experimental evidence and limited clinical experience suggest that topical steroids may reduce disease progression and the need for invasive treatment.

**Objective:**

To evaluate whether dexamethasone eye drops reduce the proportion of preterm infants with prethreshold ROP progressing to treatment-requiring type 1 ROP.

**Design, Setting, and Participants:**

The DROPROP trial was a double-masked randomized clinical trial at 6 university hospitals and 8 county hospitals in Sweden. It evaluated infants born before 30 weeks’ gestational age (GA), from 2022 to 2025, with severe ROP. Data analysis was performed from November 2025 to January 2026.

**Exposures:**

Infants were randomized to receive dexamethasone eye drops (1 mg/mL) or placebo (saline). One eye drop was administered every day or every other day for up to 12 weeks.

**Main Outcomes and Measures:**

The primary outcome was progression to type 1 ROP requiring invasive treatment. Logistic regression adjusted for GA and site was used for the primary analysis. Intention-to-treat analysis was performed. Adverse events were monitored as safety outcomes.

**Results:**

Among 100 infants, the mean (SD) GA at birth was 25.1 (1.4) weeks, 42 (42.0%) were female, and the mean (SD) birth weight was 712.9 (202.1) g. In the intention-to-treat population, type 1 ROP occurred in 10 of 50 infants (20.0%) in the dexamethasone group and 19 of 50 infants (38.0%) in the placebo group (adjusted odds ratio, 0.44; 95% CI, 0.17-1.12; *P* = .08), corresponding to a relative risk reduction of 47%. In the per-protocol population, type 1 ROP occurred in 9 of 48 infants (18.8%) in the dexamethasone group vs 19 of 49 infants (38.8%) in the placebo group (adjusted odds ratio, 0.40; 95% CI 0.15-1.05). No clinically significant differences in adverse events were observed between groups.

**Conclusions and Relevance:**

Timely administration of topical dexamethasone numerically reduced the risk of prethreshold ROP progressing to treatment-requiring type 1 ROP. Although the analysis did not reach statistical significance, these findings suggest that topical dexamethasone may be a safe, noninvasive strategy to reduce the need for invasive treatment.

**Trial Registration:**

euclinicaltrials.eu Identifier: 2023-505318-97-00

## Key Points

**Question:**

Does topical dexamethasone eye drop treatment reduce the progression of retinopathy of prematurity (ROP) to severe disease requiring invasive treatment in preterm infants?

**Findings:**

In this randomized clinical trial of 100 preterm infants with severe ROP, treatment-requiring (type 1) ROP occurred in 20.0% of infants treated with dexamethasone eye drops compared with 38.0% of those treated with placebo. However, this difference did not reach statistical significance, and no differences in adverse events were observed.

**Meaning:**

Topical dexamethasone may be a safe, noninvasive strategy to reduce progression to treatment-requiring ROP in extremely preterm infants.

## Teaser

This randomized clinical trial evaluates whether dexamethasone eye drops reduce the proportion of preterm infants with prethreshold retinopathy of prematurity progressing to treatment-requiring type 1 retinopathy of prematurity.

## Introduction

Retinopathy of prematurity (ROP) is a leading cause of preventable childhood blindness.^1,2,3,4^ In high-income countries, it primarily affects extremely preterm infants, while in lower-resource settings, more mature infants are also at risk.^5^ A global shortage of pediatric ophthalmologists limits timely treatment.^6^

ROP results from arrested retinal vascular development, followed by ischemia and inflammation that increase vascular endothelial growth factor (VEGF), which may lead to pathologic neovascularization and risk of retinal detachment.^7,8,9,10^ Type 1 ROP is severe and requires invasive treatment, whereas type 2 ROP is a prethreshold disease that requires close monitoring.^11,12^ Approximately 5% to 10% of screened infants require invasive treatment, laser therapy, or intravitreal anti-VEGF injections, both of which have limitations and adverse effects.^13,14^ Preventive, easily administered, noninvasive strategies could enable sight-saving care.

Dexamethasone, a synthetic glucocorticoid, is widely used for ocular inflammation.^15^ Pilot and retrospective studies suggest that off-label use of dexamethasone eye drops may reduce progression to treatment-requiring ROP, a finding supported by experimental models of oxygen-induced retinopathy.^16,17,18,19,20^

Given the limited evidence on the safety and efficacy of topical dexamethasone for ROP, we conducted a prospective, double-blind, multicenter, randomized clinical trial (RCT), the DROPROP trial, to assess whether dexamethasone eye drops reduce the risk of progression to type 1 ROP requiring invasive treatment in infants with severe ROP and whether their use in preterm infants is safe.

## Methods

### Study Design

This multisite, double-masked RCT was conducted at 6 university hospitals and 8 county hospitals in Sweden. Infants were enrolled between September 22, 2022, and October 23, 2025. The trial protocol has been published and is available in Supplement 1.^21^ The statistical analysis plan is available in Supplement 2. The study was approved by the Swedish Ethical Review Authority and followed Consolidated Standards of Reporting Trials (CONSORT) reporting guidelines.^22^ Parents provided written informed consent, were involved in the study design, and will be involved in reporting the study outcomes. The research nurses involved in the DROPROP trial are listed in eAppendix 1 in Supplement 3. Data analysis was performed from November 2025 to January 2026.

### Participants

Infants born before 30 weeks’ gestational age (GA) were eligible if they had prethreshold ROP defined as stage 1 or 2 without plus disease in zone I, or stage 2 or 3 in posterior zone II without plus disease. Infants were excluded if they had an ongoing external ocular infection or if the neonatologist considered eye drops unsuitable.

### Randomization and Masking

Eligible infants were identified during routine ROP screening.^9^ RetCam images (Natus Sensory) were reviewed by a steering committee of 4 pediatric ophthalmologists; at least 3 had to confirm eligibility. Infants were randomized 1:1 using a digital electronic health platform (HOPE; ADDI Medical AB). Twins were assigned to the same group. Investigators, clinicians, families, and outcome assessors were masked to group allocation; only study nurses were unmasked.

### Intervention

Infants received topical dexamethasone, 1 mg/mL (Dexafree; Théa), or placebo (preservative-free saline) in identical single-dose containers, administered by nurses. Dosing was 1 drop per eye every other day for stage 1 or 2 ROP and 1 drop per eye daily for stage 3 ROP. The intervention continued for up to 12 weeks and was adjusted, in a masked fashion, based on ROP progression or regression (eAppendix 2 in Supplement 3). The number of drops was tapered to 1 drop per eye every other day over 1 week at discontinuation. Intervention was stopped if invasive ROP treatment was initiated. In home care, caregivers recorded eye drop doses in an electronic diary.

### Outcomes

The primary outcome was the proportion of infants requiring invasive ROP treatment for type 1 ROP.^11^ RetCam images were adjudicated by the steering committee, in a masked fashion, before any invasive ROP treatment was performed; also, here, 3 of the 4 steering committee ophthalmologists had to confirm. Secondary outcomes included time to type 1 ROP, time to type 1 ROP among infants without regression, and recurrence after laser or anti-VEGF therapy. Post hoc outcomes of ROP treatment and type 1 ROP, excluding infants treated without type 1 ROP, were additionally described.

### Safety

Prespecified adverse events (AEs) included elevated intraocular pressure (IOP; >20 mm Hg), systemic growth (>1-SD difference, to capture small deviations, from the start of eye drop administration to study termination), hyperglycemia (fasting plasma glucose concentration >180 mg/dL [to convert to millimoles per liter, multiply by 0.0555]), hypoglycemia (fasting plasma glucose concentration <47 mg/dL), and pulmonary, cardiovascular, gastrointestinal, infectious, and neurologic complications. Events were graded for severity, seriousness, and causality.

### Procedures

IOP, growth parameters, vital signs, urine glucose concentration, and salivary cortisol concentration were monitored at prespecified intervals for 2 weeks after eye drop completion.

### Sample Size

Assuming type 1 ROP rates of 50% in the placebo group and 20% in the dexamethasone group, 45 infants per group provided 80% power with α = .05. Allowing for 10% attrition, 100 infants were enrolled.

### Statistical Analysis

Per protocol, participants were defined as having received eye drop doses on at least 3 days and adhered to the study eye drop administration schedule. All 100 infants were included in the intention-to-treat (ITT) population (50 in each group), and 97 were included in the per-protocol (PP) population (48 in the intervention group and 49 in the placebo group). One infant was excluded due to receiving fewer than 3 doses, 1 was excluded due to protocol violation of eye drop administration with no increase in eye drops when stage 3 ROP occurred for 2 weeks, and the guardian of 1 infant, randomized to placebo, notified the sponsor that they had provided the infant with dexamethasone eye drops to avoid invasive ROP treatment. The infant did not progress to treatment. Hence, the safety population included 51 infants in the intervention group and 49 in the placebo group.

All detailed statistical methods were documented in the statistical analysis plan (Supplement 2). All analyses were performed using SAS software version 9.4 (SAS Institute Inc).

All efficacy evaluations were adjusted for GA at birth (the most prominent risk factor) as fixed effect and site as random effect. The difference between the 2 study groups with respect to binary outcomes, such as the primary outcome, was evaluated using logistic regression, and time-to-event data were evaluated using the Cox proportional hazards model. Change in IOP was analyzed using a linear mixed-effects model with fixed effects for treatment, visit, and treatment-by-visit interaction, adjusting for baseline IOP and eye (right or left). Random intercepts for site and participant (nested within site) were included, and repeated measurements within each eye were modeled using an unstructured covariance matrix. This approach accounts for correlation between eyes within a participant and over time. Least-squares means were used to estimate treatment differences at each visit and between groups.

The primary outcome was further analyzed for the following subgroups in an exploratory manner: site, ROP severity at inclusion (stage 2 posterior zone II, stage 3), postnatal age at inclusion (median), postmenstrual age at inclusion (median), GA at birth (<25, 25-26, or ≥27 weeks), birth weight (BW; median), parenteral nutrition (<14 or ≥14 days), number of eye drops (median), number of days receiving eye drops (median), and number of eye drops per day (≤1.5, 1.5 to <2, or ≥2). An interaction term between the subgroup and the randomization group was added to the unadjusted logistic model.

The effect size for AEs was described through risk ratios, with 95% CIs, between the treatment groups.

All tests were 2-tailed. The primary analysis was the only confirmatory analysis and was considered statistically significant at *P* < .05. For the secondary end points, *P* values were evaluated following Bonferroni-Holm adjustment. All other end points were defined as exploratory and hypothesis generating rather than confirmatory; therefore, no further adjustment for type I error was applied.

## Results

### Study Population

**Figure 1.**
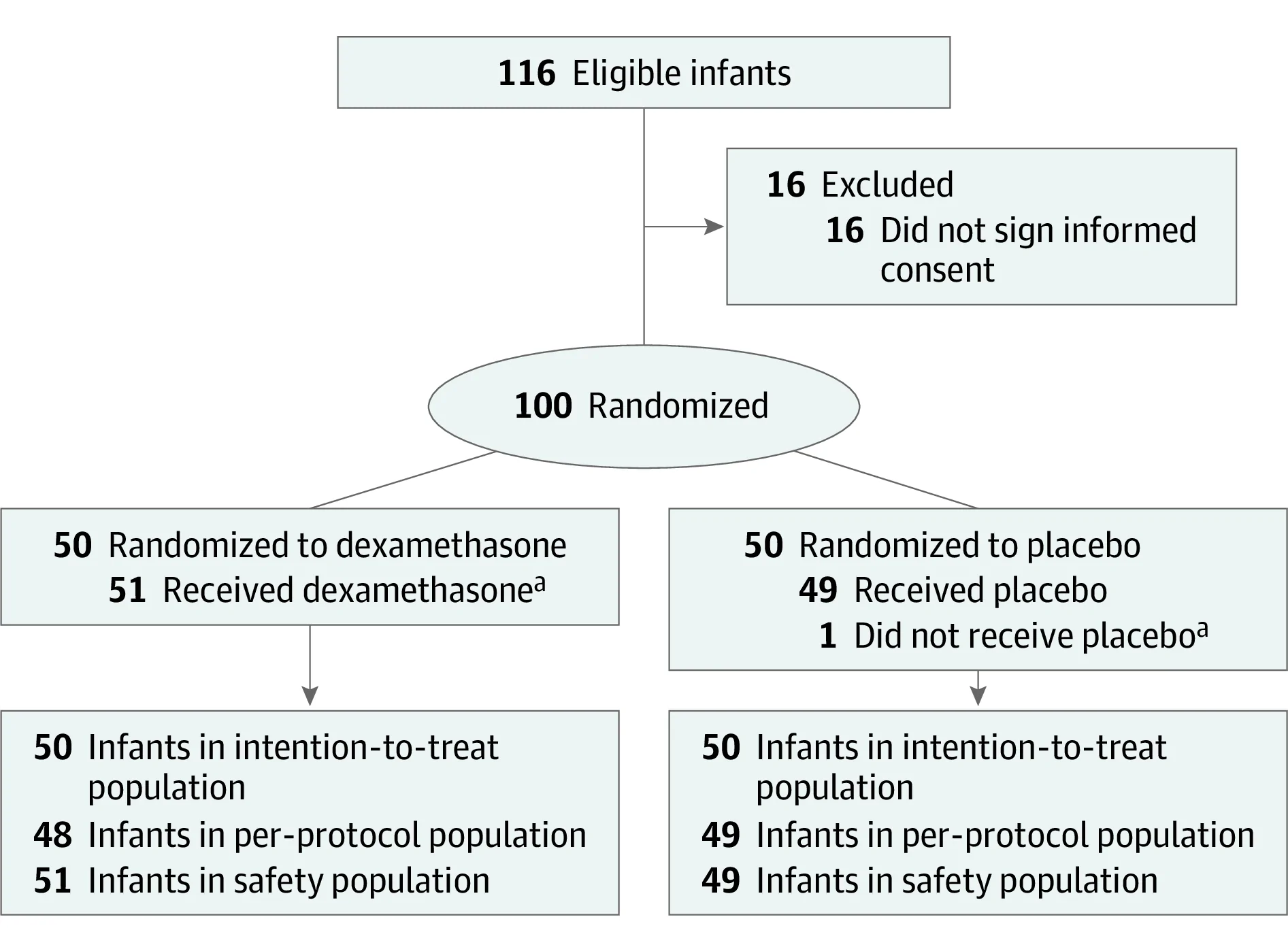
Consolidated Standards of Reporting Trials (CONSORT) Flow Diagram for the DROPROP Randomized Clinical Trial ^a^One infant received dexamethasone treatment instead of placebo.

Among 116 infants eligible for the study, parental consent was obtained for 100 infants (42 girls [42.0%] and 5 twin pairs) (Figure 1). Overall, the mean (SD) GA at birth was 25.1 (1.4) weeks, and the mean (SD) BW was 712.9 (202.1) g. In the dexamethasone and placebo groups, respectively, the mean (SD) GAs were 25.2 (1.4) and 24.9 (1.5) weeks and the mean (SD) BWs were 716.4 (196.9) and 709.3 (209.2) g (Table 1). Neonatal background and maternal history were similar between the 2 groups, although a nonsignificant overrepresentation of in vitro fertilization and preterm premature rupture of membranes was seen in the placebo group (Table 1; eTables 1 and 2 in Supplement 3). Baseline characteristics for the PP and safety populations are presented in eTables 3 and 4 in Supplement 3.

**Table 1.** Baseline Characteristics for the Intention-to-Treat Population

Characteristic	Dexamethasone (n = 50)	Placebo (n = 50)
**Infants**		
Gestational age at birth, wk		
Mean (SD)	25.2 (1.4)	24.9 (1.5)
Median (range)	25.0 (22.9-29.1)	24.6 (22.6-29.9)
Sex, No. (%)		
Female	20 (40.0)	22 (44.0)
Male	30 (60.0)	28 (56.0)
Twin, No. (%)	9 (18.0)	10 (20.0)
ROP stage at inclusion, No. (%)		
Stage 2 posterior zone II	13 (26.0)	12 (24.0)
Stage 3	37 (74.0)	38 (76.0)
Parenteral nutrition ≥14 d, No. (%)	33 (66.0)	31 (64.6)
Weight at birth, g		
Mean (SD)	716.4 (196.9)	709.3 (209.2)
Median (range)	669.5 (360.0-1414.0)	649.5 (439.0-1615.0)
Height at birth, cm		
Mean (SD)	32.2 (2.9)	31.5 (2.8)
Median (range)	32.5 (25.5-39.0)	31.0 (25.5-39.0)
Head circumference at birth, cm		
Mean (SD)	22.9 (1.9)	22.4 (2.0)
Median (range)	22.5 (20.0-30.0)	22.1 (19.0-28.5)
Postnatal age at inclusion, wk		
Mean (SD)	9.3 (2.1)	9.2 (1.5)
Median (range)	8.6 (6.1-15.0)	9.2 (6.3-13.1)
Postmenstrual age at inclusion, wk		
Mean (SD)	34.5 (2.2)	34.2 (1.7)
Median (range)	34.3 (31.3-41.3)	33.9 (30.9-39.7)
**Mothers**		
Mother’s age, y		
Mean (SD)	33.0 (5.1)	31.7 (4.8)
Median (range)	34.0 (22.0-42.0)	31.0 (21.0-42.0)
Any chronic disease, No. (%)	13 (26.0)	12 (24.0)
Parity, No.		
Mean (SD)	1.6 (1.1)	1.6 (0.9)
Median (range)	1.0 (1.0-6.0)	1.0 (0.0-6.0)
In vitro fertilization, No. (%)	8 (16.0)	13 (28.9)

Abbreviation: ROP, retinopathy of prematurity.

The median (range) eye drop duration was 5.3 (0.4-12.7) weeks (eTable 5 in Supplement 3). Seventeen infants received the intervention for 12 weeks, and 2 infants received it for less than 1 week. The mean (SD) number of investigational eye drops received was 71 (49), and 45 of 50 infants (90.0%) received 1 drop per eye per day sometime during the study intervention.

### Primary Outcome

**Figure 2.**
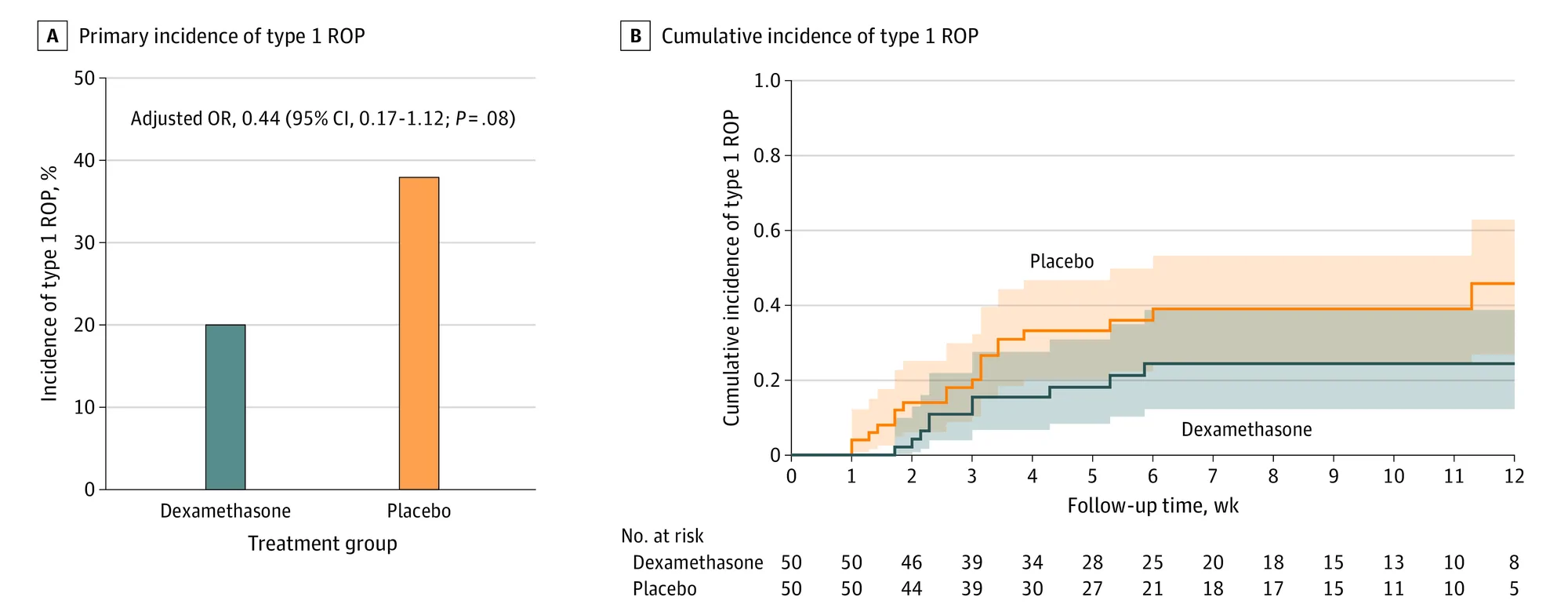
Bar Graph of the Primary Incidence of Type 1 Retinopathy of Prematurity (ROP) and Kaplan-Meier Plot of the Cumulative Incidence of Type 1 ROP in the Intention-to-Treat Population A, Bar graph shows the primary incidence of type 1 ROP for the dexamethasone and placebo groups. The adjusted odds ratio (OR) with 95% CI presented for the incidence of type 1 ROP in the dexamethasone group vs the placebo group was adjusted for gestational age (fixed effect) and site (random effect). B, Kaplan-Meier plot shows the cumulative incidence of type 1 ROP. Shaded area indicates 95% CI.

In the ITT population, 29 of 100 infants were treated with laser or anti-VEGF for type 1 ROP: 10 of 50 infants (20.0%) in the dexamethasone group and 19 of 50 infants (38.0%) in the placebo group. The unadjusted odds ratio (OR) was 0.41 (95% CI, 0.17-1.00). The primary analysis was adjusted for GA and site, resulting in an OR of 0.44 (95% CI, 0.17-1.12; *P* = .08), corresponding to a statistically nonsignificant relative risk reduction of 47% (Figure 2A and Table 2).

**Table 2.** Analyses of Secondary Outcomes in the Intention-to-Treat Population

Outcome	Dexamethasone		Placebo		Unadjusted analyses, effect size (95% CI)^a^	Adjusted analyses^a^	
	No.	Events, No. (%)	No.	Events, No. (%)		Effect size (95% CI)	*P* value
Primary analysis							
Incidence of type 1 ROP during follow-up^b^	50	10 (20.0)	50	19 (38.0)	0.41 (0.17-1.00)	0.44 (0.17-1.12)	.08
Post hoc sensitivity analyses							
Incidence of ROP treatment during follow-up^b^	50	15 (30.0)	50	21 (42.0)	0.59 (0.26-1.35)	0.63 (0.27-1.48)	.28^c^
Incidence of type 1 ROP during follow-up, excluding infants treated without type 1 ROP^b^	45	10 (22.2)	48	19 (39.6)	0.44 (0.18-1.08)	0.50 (0.19-1.28)	.15^c^
Secondary analyses							
Time to type 1 ROP (laser, n = 15; anti-VEGF, n = 14) during follow-up^d^	50	10 (20.0)	50	19 (38.0)	0.52 (0.24-1.11)	0.61 (0.28-1.34)	.22^c^
Time to ROP treatment during follow-up^d^	50	15 (30.0)	50	21 (42.0)	0.69 (0.36-1.34)	0.78 (0.40-1.53)	.47^c^
Proportion of infants with any recurrence after laser (n = 2) or anti-VEGF (n = 7) treatment during follow-up^b^	50	4 (8.0)	50	5 (10.0)	0.78 (0.20-3.10)	1.04 (0.24-4.54)	.96^c^

Abbreviations: ROP, retinopathy of prematurity.

^a^ The unadjusted analyses were considered supportive; the adjusted analyses were considered the main analyses and were adjusted for gestational age (fixed) and site (random).

^b^ Binary outcomes, with analyses performed using logistic regression. Effect sizes present the odds ratio with 95% CI.

^c^ Nonsignificant following Bonferroni-Holm adjustment.

^d^ Time-to-event outcomes, with analyses performed using Cox regression. Effect sizes present the hazard ratio with 95% CI.

In the PP population, type 1 ROP occurred in 9 of 48 infants (18.8%) in the dexamethasone group and 19 of 49 infants (38.8%) in the placebo group. The unadjusted OR was 0.36 (95% CI, 0.14-0.92). Adjusted for GA and site, the OR was 0.40 (95% CI, 0.15-1.05) (eTable 6 in Supplement 3).

An additional 7 infants with prethreshold ROP underwent invasive treatment at the discretion of local ophthalmologists, despite guidance from the steering committee that these infants did not meet the established treatment criteria (5 in the dexamethasone group and 2 in the placebo group), as shown in Table 2. In 5 of these cases, the reason for treatment was logistics, eg, it was done in conjunction with surgery and anesthesia for other reasons, the treating physician was available, or treatment was done prior to transfer to the regional hospital. In 2 cases, 1 in each group, there were local neovascularizations in zone III in 1 clock hour.

Subgroup analyses of the primary efficacy outcome showed that the effect of dexamethasone eye drops was numerically greater in those with BW greater than 660 g (OR, 0.23; 95% CI, 0.06-0.94) and in infants who received parenteral nutrition for less than 14 days (OR, 0.04; 95% CI, 0.00-0.84) (eTable 7 in Supplement 3).

### Secondary Outcomes

Secondary analyses are presented in Table 2 for the ITT population and eTable 8 in Supplement 3 for the PP population. In the ITT population, there was no difference in the time from randomization to type 1 ROP or to the end of eye drop treatment between the 2 groups (adjusted hazard ratio, 0.61; 95% CI, 0.28-1.34) (Figure 2B).

Nine infants had ROP recurrence, ie, at least 2 invasive ROP treatment sessions, including 2 infants after laser treatment and 7 infants after anti-VEGF treatment. Four of these infants were in the dexamethasone group vs 5 in the placebo group.

### Safety Evaluations

The risk ratios for AEs overall and AEs of special interest were either close to 1 or had a very wide 95% CI. There was no clinically significant difference in IOP between infants receiving dexamethasone and those receiving placebo, nor did we observe any increase in IOP during dexamethasone treatment compared with baseline in any of the groups (eFigure 1 in Supplement 3). In the dexamethasone group, 13 infants (25.5%) had at least 1 IOP measurement higher than 20 mm Hg, of whom 11 were considered possibly eye drop related; 10 infants (20.4%) in the placebo group had at least 1 IOP measurement higher than 20 mm Hg (Table 3). In 8 infants, the elevated IOP was attributed to eyelid pressure. Four infants in the dexamethasone group were reported to have a reduction in weight SD scores greater than 1 from inclusion to study end, possibly treatment related in 3 of these 4 infants, and 1 infant in the placebo group had a reduction of at least 1 SD in length (Table 3; eFigures 2-4 in Supplement 3). IOP and growth SD score were the only reported AEs possibly related in the study.

**Table 3.** Adverse Events (AEs) in the Safety Population

AE	Dexamethasone (n = 51)		Placebo (n = 49)		Dexamethasone vs placebo, risk ratio (95% CI)^a^
	Events, No.	Infants, No. (%)	Events, No.	Infants, No. (%)	
Any AE	61	36 (70.6)	55	36 (73.5)	0.96 (0.75-1.23)
Any SAE	41	33 (64.7)	44	36 (73.5)	0.88 (0.68-1.15)
Any treatment-related AE	14	14 (27.5)	10	10 (20.4)	1.35 (0.66-2.74)
Any treatment-related SAE	0	0	0	0	NA
AEs of special interest					
Abnormal growth	4	4 (7.8)	1	1 (2.0)	3.84 (0.45-33.19)
Bronchopulmonary dysplasia	33	33 (64.7)	33	33 (67.3)	0.96 (0.73-1.27)
Eye pressure >20 mm Hg	16	13 (25.5)	11	10 (20.4)	1.25 (0.60-3.04)
Ileus	0	0	2	2 (4.1)	NA
Enterostomy	1	1 (2.0)	2	2 (4.1)	0.48 (0.04-5.13)
Hypoglycemia	1	1 (2.0)	2	2 (4.1)	0.48 (0.04-5.13)
Hypotension	0	0	1	1 (2.0)	NA
Surgical patent ductus arteriosus	2	2 (3.9)	0	0	NA
Pulmonary hypertension	0	0	1	1 (2.0)	NA
Suspected sepsis	6	6 (11.8)	3	3 (6.1)	1.92 (0.51-7.26)
Confirmed sepsis	3	3 (5.9)	2	2 (4.1)	1.44 (0.25-8.26)

Abbreviations: NA, not applicable; SAE, serious AEs.

^a^ Estimated using exact Poisson limits.

The total number of AEs was similar between the 2 groups: 61 events among 36 infants (70.6%) in the dexamethasone group and 55 among 36 infants (73.5%) in the placebo group. Serious AEs were observed in 33 infants (64.7%) in the dexamethasone group vs 36 infants (73.5%) in the placebo group. Confirmed sepsis was defined as clinical symptoms with a positive blood culture result; in cases of *Staphylococcus epidermidis* or coagulase-negative staphylococci, an additional C-reactive protein level greater than 2 mg/dL (to convert to milligrams per liter, multiply by 10) was required. With this strict definition, there were 3 cases in the dexamethasone group and 2 in the placebo group (Table 3).

Salivary cortisol measurements were lower during treatment in the dexamethasone group and normalized after treatment cessation (eFigure 5 in Supplement 3).

## Discussion

In this RCT, topical dexamethasone numerically reduced the risk of progression from prethreshold ROP to treatment-requiring type 1 ROP, although the observed 47% relative risk reduction (OR, 0.44; 95% CI, 0.17-1.12; *P* = .08) did not reach statistical significance and thus the study hypothesis could not be confirmed. However, based on the clinically meaningful effect size, our findings suggest that topical dexamethasone may likely be useful as a noninvasive adjunct to reduce progression to invasive ROP treatment in preterm infants. Larger, adequately powered RCTs are recommended before routine clinical implementation can be considered to statistically confirm our findings.

Dexamethasone is widely used to treat ocular inflammation and corneal neovascularization. In adults, poor corneal permeability limits the efficacy of topical dexamethasone in retinal disorders; however, in newborns, an immature epithelial barrier may enhance ocular absorption.^23,24^ The mechanisms by which dexamethasone may limit ROP progression extend beyond its anti-inflammatory effects. ROP is preceded by disordered oxygen exposure and bioenergetic failure related to insufficient nutrient utilization. In a mouse model of oxygen-induced retinopathy, topical dexamethasone reduced inflammation and angiogenesis, improved oxidative phosphorylation, and decreased neovascularization by 30% when administered before peak disease. Blocking mitochondrial adenosine triphosphate production reversed these effects, suggesting that promoting mitochondrial energy generation may prevent ROP progression.^17^ The role of bioenergetic stress and mitochondrial dysfunction in ROP is supported by a recent study showing that a rapid early postnatal increase in fibroblast growth factor 21, a metabolic stress hormone, was associated with later severe ROP in extremely preterm infants.^25^

Research in preterm infants is challenging due to their vulnerability and critical illness. Yet, it is essential, as insufficient pediatric studies and widespread off-label medication use have historically contributed to preventable clinical misadventures.^26,27,28^ In this RCT, AEs were our primary concern, given the limited evidence of the safety of off-label use of topical dexamethasone in preterm infants. Topical corticosteroids can be systemically absorbed, potentially leading to growth suppression or adrenal suppression, altered glucose metabolism, or increased risk of infection, although these effects are poorly characterized in preterm infants.^15,29,30,31^ In our trial, there was no clinically significant difference in AEs between the groups during the trial period. No infant in the trial demonstrated clinical evidence of adrenal suppression. As anticipated, salivary cortisol concentrations were reduced during treatment and returned to baseline after discontinuation. In prior studies, up to two-thirds of infants receiving standard glucocorticoid protocols after congenital cataract surgery showed adrenal suppression, which was dose and duration dependent.^32^ During the study period, there was no difference in episodes of hyperglycemia between the groups. A threshold of more than 1 SD was prespecified to detect early growth deviations, whereas a deviation of more than 2 SDs represents clinically significant growth failure, as it identifies infants at substantially increased risk for adverse outcomes, including mortality and major neonatal morbidities.^33^ Two infants in the dexamethasone group had deviated by more than 2 SDs in weight during the intervention period. One infant underwent surgery for ileus with enterostomy and received parenteral nutrition for 2.8 months; this deviation was considered unrelated to the study drug. The other infant experienced feeding difficulties requiring more than 1.5 months of parenteral nutrition due to subglottic stenosis and tracheostomy, and a possible association with the study drug could not be excluded. In the DROPROP trial, the intervention was limited to 1 drop per eye daily for up to 12 weeks due to the risk of systemic effects, and close monitoring during dexamethasone eye drop treatment and long-term follow-up of infants cannot be overemphasized.

Potential ocular adverse effects, mainly reported in adults, include elevated IOP, glaucoma, and cataract.^15^ We found no difference in IOP levels between the groups, with 22% in the dexamethasone group and 20% in the placebo group showing a possibly treatment-related IOP greater than 20 mm Hg. Two pilot studies and a retrospective comparison suggested that dexamethasone eye drops may help prevent ROP.^16,17,18^ However, these studies did not routinely collect or systematically evaluate safety outcomes during off-label use. Participants in the DROPROP trial will undergo long-term follow-up at ages 2.5 and 6.5 years to evaluate ocular and visual outcomes and the potential for late adverse effects.

We found a 47% decrease in progression from prethreshold ROP to severe ROP requiring invasive treatment, which did not reach statistical significance and was somewhat lower than the 60% effect assumed in the study’s sample size calculations. Despite the lack of statistical significance, the observed effect size, together with noninvasive administration, low cost, and a clinically acceptable safety profile, suggests that topical dexamethasone might become a valuable tool, potentially reducing exposure to general anesthesia, destructive laser therapy, and anti-VEGF injections.^34,35^ Given the association of invasive ROP treatment with long-term visual impairment and the potential neurodevelopmental risks of anesthesia and anti-VEGF therapy, as well as retinal damage from laser treatment, safer and more accessible therapies are needed.^13,14,36,37,38^ The intervention had no effect on treatment timing or retreatment rates. Interestingly, there was a numerically greater effect of dexamethasone eye drops for healthier infants (parenteral nutrition <14 days) and heavier infants (BW >660 g). These findings indicate that in infants in whom metabolic status is less important as a risk factor for ROP, the intervention may have greater effects. Further studies are needed, including more infants and those representing the populations that currently account for the large increase in blinding ROP in low- and middle-income countries. Many of these infants have higher GA and BW and more mature retinal vascularization at birth than infants with severe ROP in high-income countries with more advanced neonatal care. Uncontrolled oxygen supply destroys retinal vessels, leading to severe ROP, often in settings with limited screening and treatment facilities. If easily administered noninvasive dexamethasone eye drops efficiently prevent ROP progression in these populations, sight could be saved. Studies in these populations are warranted.

### Strengths and Limitations

This trial has several strengths. It was a multisite study and the first RCT to date, to our knowledge, assessing topical dexamethasone in preterm infants with severe ROP. The lack of a prior RCT was verified in a database search in ClinicalTrials.gov and in the EudraCT database in November 2025, with the search terms *RCT*, *retinopathy of prematurity*, and *dexamethasone eye drops*. A professional steering group had to confirm inclusion and type 1 criteria by reviewing retinal images to ensure data quality. Clinicians, families, and outcome assessors were masked to group allocation, reducing the risk of bias arising in the clinical management or data collection. Primary outcome data were available for all infants.

The trial also has several limitations. The anticipated treatment effect of dexamethasone was overestimated in the sample size calculation (60% assumed vs 47% observed), likely due to the effect observed in the pilot unmasked study with a higher-dose regimen.^18^ Additionally, the event rate in the placebo group was lower than expected (38% observed vs 50% estimated), potentially reflecting temporal changes in neonatal care that may have influenced baseline risk.^39^ Together, these factors may have reduced the study’s statistical power to detect a significant difference between groups.

It should also be noted that 7 infants were treated despite the steering committee not verifying type 1 ROP (5 in the dexamethasone group and 2 in the placebo group). They were mainly treated for logistical reasons, and if included in the analysis, the effect of dexamethasone eye drops would be diminished.

## Conclusions

Although not statistically significant, the findings from this RCT suggest a potential benefit of topical dexamethasone in preventing the need for invasive ROP treatment. Larger RCTs are needed before adoption into national guidelines to ensure optimal care and confirmed safety and to identify potential subgroups of infants who may benefit most. Nonetheless, the study highlights the potential to reduce the risk of visual impairment in preterm infants, with meaningful implications for long-term quality of life.

## Notes

Supplement 1. Trial Protocol

Supplement 2. Statistical Analysis Plan

Supplement 3. **eAppendix 1.** Research Nurses Involved in the DROPROP Study **eAppendix 2.** DROPROP Dose-Adjusting Scheme **eFigure 1.** Box Plot of IOP Over Time (Safety Population) **eFigure 2.** Box Plots of Weight (g) and SDS Over Time (Safety Population) **eFigure 3.** Box Plots of Height (cm) and SDS Over Time (Safety Population) **eFigure 4.** Box Plots of Head Circumference (cm) and SDS Over Time (Safety Population) **eFigure 5.** Box Plot of Salivary Cortisol Concentration (ITT Population) **eTable 1.** Infants’ Medical History (ITT Population) **eTable 2.** Mothers’ Pregnancy Data and Complications (ITT Population) **eTable 3.** Baseline Characteristics (PP Population) **eTable 4.** Baseline Characteristics (Safety Population) **eTable 5.** Exposure (Safety Population) **eTable 6.** Robustness Analyses of the Primary Outcome (PP Population) **eTable 7.** Subgroup Analysis of the Primary Outcome (ITT Population) **eTable 8.** Analyses of Secondary Outcomes (PP Population)

Supplement 4. Members of the DROPROP Collaboration Group

Supplement 5. Data Sharing Statement
