# Supplementary material for: Dexamethasone Eye Drops to Prevent Treatment-Requiring Retinopathy of Prematurity: The DROPROP Randomized Clinical Trial
Source: JAMA Pediatr. 2026 Aug 3:e263247. Online ahead of print. doi: 10.1001/jamapediatrics.2026.3247 (PMC13434969; doi:10.1001/jamapediatrics.2026.3247)
Supplement: Supplementary file 1 [file jamapediatr-e263247-s001.pdf]

CLINICAL STUDY PROTOCOL

A Randomized Intervention, Multi-Center Study to Determine the Role of Dexamethasone eye drops against proliferative retinopathy of prematurity

EudraCT# 2020-004933-19

Date: 251008

Sponsor and Principal Investigator: Ann Hellström MD, PhD.[1]
Tel: +46 (0)768 979196
Fax: +46 (0)31 848952

Co-investigators Ophthalmology:
Jenny Wallander MD[3]
Ylva Friberg Riad MD[4]
Eva Larsson MD, PhD[5]
Karin Hochard MD[6]
Stefan Löfgren MD, PhD[7]
Evangelos Tsigkoulis, MD[8]
Aikaterini Massou, MD[9]
Sofie Eriksson, MD, PhD[10]
Hanna Åkerblom, MD, PhD [11]

**Co-investigators Neonatology:**	[1]Liv Södermark, Gbg
	[2]Ingrid Pupp, Lund
	[3]Fredrik Ingemansson, Jönköping
	[4]Ivo Krassimirov Kaltchev, Skövde
	[5]Erik Normann, Uppsala
	[6]Magnus Domellöf, Umeå
	[7]Nikica Tomasic, Stockholm
	[8]Per Odelberg-Johnson, Linköping
	[9]Uwe Ewald, Gävle
	[10]Eva Albinsson, Karlstad
	[11]Peter Jotorp, Västerås
**Scientific Adviser**:	Lois Smith MD, PhD
	David Ley MD, PhD[2]
**Clinics:**	[1]Drottning Silvias Barnsjukhus, Göteborg
	[2]Skånes Universitetssjukhus, Lund
	[3]Ryhovs Sjukhus, Jönköping
	[4]Skaraborgs Sjukhus, Skövde
	[5]Akademiska Sjukhuset, Uppsala
	[6]Norrlands Universitetssjukhus, Umeå
	[7]St Eriks Eye Hospital och Karolinska Universitetssjukhuset, Stockholm
	[8]Universitetssjukhuset Linköping, Linköping
	[9]Gävle Sjukhus, Gävle
	[10] Centralsjukhuset Karlstad
	[11] Västmanlands Sjukhus, Västerås
**Producer of Drug:**	**THEA Pharmaceuticals**
	Storgatan 55
	70363 Örebro
	Sweden
	T M +46705154632

Nordic Marketing Manager

Study nurses: See amendment

Monitor: Carola Pfeiffer Mosesson

Institutionen för neurovetenskap och fysiologi, Göteborgs Universitet

This clinical study will be conducted, and essential study documentation archived, in compliance with requirements of the ICH Guidelines for Good Clinical Practice and Swedish laws

Content

PROTOCOL SYNOPSIS **6**
LIST OF ABBREVIATIONS AND TERMS **7**
1. BACKGROUND AND RATIONALE **10**
1.1. GENERAL INTRODUCTION 10
1.2. RATIONALE AND JUSTIFICATION FOR THE STUDY 10
2. HYPOTHESIS AND OBJECTIVES **11**
2.1. HYPOTHESIS 11
2.2. PRIMARY OBJECTIVE 11
2.3. SECONDARY OBJECTIVES 11
2.4. POTENTIAL RISKS AND BENEFITS 12
2.4.1. POTENTIAL RISKS 12
2.4.2. POTENTIAL BENEFITS 13
3. STUDY POPULATION **13**
3.1. INCLUSION CRITERIA 13
3.2. EXCLUSION CRITERIA 13
3.3. WITHDRAWAL CRITERIA 14
3.4. SUBJECT REPLACEMENT 14
4. STUDY SCHEDULE **14**
5. STUDY DESIGN **16**
6. METHODS AND ASSESSMENTS **16**
6.1. RANDOMIZATION AND BLINDING 16
6.2. STUDY VISITS AND PROCEDURES 16

6.2.1. Treatment Phase Visits and Procedures ... 16
6.2.2. Treatment Phase Study Termination Visit ... 19
6.2.3. Extension Study Follow-up and Procedures ... 19

7. TRIAL MATERIALS ... 20

7.1. Trial Products ... 20
7.2. Storage and Accountability ... 21

8. STUDY TREATMENT ... 21

8.1. Rationale for Selection of Dose ... 21
8.2. Study Treatment Formulation ... 21
8.3. Study Treatment Administration ... 21
8.4. Specific Restrictions/Requirements ... 22
8.5. Blinding ... 22
8.6. Concomitant Therapy ... 23

9. SAFETY MEASUREMENTS ... 23

9.1. Definitions ... 23
9.1.1. Adverse Events ... 23
9.1.2. Serious Adverse Events ... 23
9.2. Collecting, Recording and Reporting Adverse Events ... 24
9.2.1. Adverse Events ... 24
9.2.2. Serious Adverse Events ... 25
9.3. Safety Monitoring Plan ... 26
9.4. Training ... 26

10. DATA MANAGEMENT ... 26

10.1. eCase Report Forms ... 26
10.2. Data Quality Assurance ... 27
10.3. Data Entry and Storage ... 27

11. SAMPLE SIZE AND STATISTICAL METHODS ... 27

11.1. Determination of Sample Size ... 27
11.2. Statistical and Analytical Plan ... 28
11.2.1. Analysis Populations ... 28
11.2.2. General Considerations ... 28
11.2.3. Primary Efficacy Analysis ... 29
11.2.4. Secondary Efficacy Analyses ... 30
11.2.5. Safety Analyses ... 30
11.2.6. Data Monitoring Committee ... 30

12. ETHICAL CONSIDERATIONS ... 31

12.1. Independent Ethics Committee ... 31
12.2. Informed Consent Form ... 31
12.3. Confidentiality of Data and Patient Records ... 32
12.4. Biobank ... 32

13. PUBLICATIONS ... 32

14. RETENTION OF TRIAL DOCUMENTS ... 32

14.1. Study Data ... 32
14.2. Protocol Amendments ... 33

15. REFERENCES ... 34

PROTOCOL SYNOPSIS

Protocol Title: A Randomized Intervention, Multi-Center Study to Determine the Role of Dexamethasone eye drops against proliferative retinopathy of prematurity

Protocol Number: DROPROP 2020-1

Number of Subjects: 50 subjects receiving Dexamethason eyedrops and 50 subjects receiving placebo.

Subject Population: 100 premature male/female infants, born before gestational age 30 weeks + 0 days.

Dosage: The eye drops will be given at a dose of 1 drop every second day to one drop daily in affected eyes.

Maximal treatment duration is 12 weeks.

If ROP Type 1 develops the dose is increased to 1 x 3 daily until treatment within 72 hours and after treatment 2 drops daily for 5 days and one drop daily for two days.

Study Duration: Treatment Phase (main) study will last for maximum 12 weeks between approximately 34 to 40 postmenstrual weeks. In the Extension study infants will be followed up at 2.5 and 6.5 years of age.

Primary Objective: To reduce number of infants needing conventional treatment for severe ROP

Primary Outcome: The proportion of infants in each group with ROP progression to Type 1 ROP needing conventional treatment.

Secondary Outcomes: 1) Time from detection of proliferative ROP to Type 1 ROP.

2) Recurrences after laser/Anti-VEGF treatment in dexamethasone treatment and placebo arm.

3) Retinal morphology at 40 weeks PMA and at 2.5 and 6.5 years with optical coherence tomography (OCT).

4) Measurements of intraocular pressure before treatment at 1 and 2 weeks after start of treatment, after completed treatment and at 2.5 and 6.5 years of age.

5) Visual outcome and refractive errors at 2.5 and 6.5 years of age.

6) To analyse leftovers of blood from the clinical blood sampling with regard to selected biomarkers and their relation to ROP development and treatment effect.

Safety Outcomes: Adverse events, clinical chemistry, retinal exam, physical examination, vital signs.

Study Administrative Structure

Sponsor and Principal Investigator:

Ann Hellström MD, PhD. Professor
Inst för neurovetenskap/fysiologi
Göteborg Universitet
Drottning Silvias barn och ungdomssjukhus/Östra
SE – 416 85 GÖTEBORG
Tel: +46 (0)31-3434722
Fax: +46 (0)31-3435771
E-mail : ann.hellstrom@medfak.gu.se

Producer of Drug:

THEA Pharmaceuticals
Nordic Marketing Manager
Storgatan 55
SE-70363 ÖREBRO
Tel: +46 (0) 705154632

Laboratory Cortisol:

Charlotte Becker and Anders Blomgren
Specialkemi, våning 3
Klinisk Kemi och Farmakologi
221 85 Lund
Tel: +46 46 173460

List of Abbreviations and Terms

**AE**	Adverse Event
**BPD**	Bronchopulmonary Dysplasia

**BW**	Birth Weight
**CI**	Confidence Interval
**CNS**	Central Nervous System
**CRF**	Case Report Form
**CV**	Curriculum Vitae
**DCF**	Data Clarification Form
**DMC**	Data Monitoring Committee
**DTI**	DiffusionTensor Imaging
**ECG**	Electrocardiografi
**EPN**	Etikprövningsmyndigheten
**GA**	Gestational Age
**GCP**	Good Clinical Practice
**GMP**	Good Manufacturing Practices
**GW**	Gestational Week
**HC**	Head Circumference
**ICH**	International Conference on Harmonization
**ITT**	Intention to Treat
**IVH**	Intraventricular hemorrhage
**MRI**	Magnetic Resonance Imaging
**NEC**	Necrotizing Enterocolitis
**NICU**	Neonatal Intensive Care Unit
**PDA**	Patent Ductus Arteriosus
**PMA**	Postmenstrual Age
**PP**	Per Protocol
**QA**	Quality Assurance
**ROP**	Retinopathy of Prematurity
**SAE**	Serious Adverse Event
**SD**	Standard Deviation
**SOP**	Standard Operation Procedures
**TMF**	Trial Master File
**TPN**	Total Parental Nutrition

**US**	Ultrasound
**VLBW**	Very Low Birth Weight

1. Background and Rationale

1.1. General Introduction

Retinopathy of prematurity (ROP) affects primarily extremely preterm infants and is characterised by a first phase of arrested retinal vascularization after birth. In severe cases ischemia and inflammation in the peripheral avascular retina cause vascular endothelial growth factor (VEGF) induced pathologic neovascularisation and, in the worst case, retinal detachment and blindness. To prevent blindness approximately 40-50 infants per year are treated with retinal laser photocoagulation in general anaesthesia or in a few cases with intravitreal anti-VEGF therapy (1). Indications for treatment are regulated by international guidelines (2) based on an international classification of ROP (3). When treatment warranting ROP has been diagnosed, treatment should be performed within 72 hours. A minor portion of infants are treated for central ROP (zone I). These cases carry the greatest risk of rapid deterioration and so called acute posterior ROP (APROP). In the majority of infants, treatment is performed in the middle zone (zone II) while indications for treatment are lacking for zone III disease.

Dexamethasone is a synthetic glucocorticoid commonly used on the ocular surface or intraocular to treat inflammation and neovascularisation (4, 5). Intravenous or oral dexamethasone is used to wean preterm infants from the ventilator. Used to prevent bronchopulmonary dysplasia an initial high and thereafter tapering dose 0.5mg/kg per day of dexamethasone the first day of life resulted in increased risk of cerebral palsy and impaired motor and cognitive development. However, treatment after one week of age and lower doses have not been shown to have the same adverse effects (6, 7).

Of infants with neovascular ROP approximately 50% will eventually need treatment with laser and/or anti-VEGF. If treatment with dexamethasone eye drops is efficient in preventing progression to treatment warranting ROP, infants may escape general anaesthesia. Surgery in general anaesthesia in preterm infants is associated with reduced cerebral white matter volumes and worse neurodevelopmental outcome at two years of age (8). Retinal laser therapy destroys large parts of the peripheral retina and after intravitreal injection, anti-VEGF agents enter the blood stream where they suppress VEGF with unknown long term effects (9).

1.2. Rationale and Justification for the Study

ROP treatment generally takes place at several weeks of age at a postmenstrual age (PMA) >34 weeks. To reduce inflammation before laser therapy, dexamethasone eye drops have been given to infants in Lund, Skåne University Hospital, Sweden. During 2020 only one out of 75 screened infants, 1.3%, have developed ROP needing treatment (Type 1) after start of Dexafree® drops according to the proposed scheme in the present study. This should be compared with a treatment rate of 6% over the previous years.

We intend to perform a randomised, blinded, controlled, multicentre trial where preterm infants with ROP and retinal neovascularisation or haemorrhages indicating the presence of neovascularisations in zone II are randomised to either receive eye drops with Dexamethasone or physiological saline (placebo) for up to 12 weeks. The study will include a Treatment Phase (up to 40 weeks of PMA or end of ROP screening) and a Follow-up Phase (at 2.5 and 6.5 years of age).

2. Hypothesis and Objectives

2.1. Hypothesis

The hypothesis is that the randomized group preventatively treated with Dexamethasone eye drops will reduce the number of infants needing ROP treatment (Type 1) with laser and/or Anti-VEGF by 55% compared to the Placebo group.

2.2. Primary Objective

The primary objective in this study is to examine whether preventative treatment with topical Dexamethasone treatment may reduce the proportion of infants who need conventional ROP treatment in comparison to Placebo arm.

The primary objective will be evaluated on the primary outcome that is:

- The proportion of infants with ROP progression to Type 1 ROP needing conventional treatment in each study arm.

2.3. Secondary Objectives

The secondary objectives are:

1) To study time from detection of proliferative ROP to Type 1 ROP in Dexamethasone treatment and Placebo arm and will be evaluated by following secondary outcomes:
    a. Time from randomization to Type 1 ROP or end of treatment phase study (all infants)
    b. Time from randomization to Type 1 ROP (infants without regression)
2) To study recurrences after laser/Anti-VEGF treatment in Dexamethasone treatment and Placebo arm and will be evaluated by following secondary outcome:
    a. The proportion of infants with any recurrences after laser/Anti-VEGF treatment (all infants)
    b. The number of recurrences per infant per follow-up time after laser/Anti-VEGF treatment (all infants)
    c. The proportion of infants with any recurrences after laser/Anti-VEGF treatment (infants progressed to Type 1 ROP)
    d. The number of recurrences per infant per follow-up time after laser/Anti-VEGF treatment (infants progressed to Type 1 ROP)
3) To study retinal morphology at 40 weeks PMA and at 2.5 and 6.5 years with optical coherence tomography (OCT) in Dexamethasone treatment and Placebo arm and will be evaluated by:
    a. Optic disc, cup and rim area at 40 weeks PMA and 2.5 and 6.5 years of age
    b. Retinal nerve fiber thickness at 40 weeks PMA and 2.5 and 6.5 years of age
    c. Macular thickness at 40 weeks PMA and 2.5 and 6.5 years of age

4) To study measurements of intraocular pressure before treatment at 1 and 2 weeks after start of treatment, after completed treatment and at 2.5 and 6.5 years will be evaluated by:
    a. Change in IOP (mmHg) from before treatment, 1-2 weeks after treatment, and after end of treatment
5) To study visual outcome and refractive errors at 2.5 and 6.5 years of age in Dexamethasone treatment and Placebo arm and will be evaluated by:
    a. Visual acuity at 2.5 (Fix and follow) and 6.5 years (KM or HVOT) of age
    b. Refraction at 2.5 and 6.5 years of age
    c. Orthoptic status including motility, stereovision, presence of nystagmus and strabismus at 2.5 and 6.5 years of age
6) To evaluate safety and tolerability of the treatment through adverse events, clinical chemistry, retinal exam, intraocular pressure, physical examination, MRI of the brain, body composition with PeaPod (Gothenburg) and vital signs.
7) To analyse leftovers of blood from clinical blood sampling with regard to selected biomarkers and their relation to ROP development and treatment effect.

2.4. Potential Risks and Benefits

2.4.1. Potential Risks

Both general anesthesia and anti-VEGF-treatment are suspected to affect brain development. Laser treatment of the retina destructs the peripheral retina. Our judgement is that the risk of local eye drops with a low dose of dexametasone for a limited time period (one to 12 weeks) is of less risk to the infant as compared to general anesthesia, laser and/or anti-VEGF treatment.

The volume of a commercial eye drop is 50-75 µl (10) and the concentration of dexamethasone in Dexafree® eye drops is 1 mg/ml. The maximal dose given at an age when almost all infants have a body weight over 2 kg (11) will be 50-75 µl x 2 equivalent to 0.1-0.15 mg/day of which the major part will be wiped off the skin. To reduce systemic absorption of the medication we will inform caregivers to apply the tissue press method (2-3 sec) to reduce medication absorption in the nasal mucosa. Treatment will be given at a several weeks of age and during a limited period of time. We believe that systemic adverse effects are unlikely. Intravenous or oral dexamethasone is used to wean preterm infants from the ventilator. Used to prevent bronchopulmonary dysplasia an initial high and thereafter tapering dose 0.5mg/kg per day of dexamethasone the first day of life resulted in increased risk of cerebral palsy and impaired motor and cognitive development. However, treatment after one week of age and lower doses have not been shown to have the same adverse effects and the dose we are exposing the infant to later in life is 0.1-0.15 mg/day (6, 7).

Common ocular side effects of short-term topical dexamethasone are local discomfort during application and increased intraocular pressure first noticed during the first two weeks of treatment (according to the product information of Dexafree®). We will therefore measure the intraocular pressure before and after one and two weeks of treatment and after completed treatment (note if baby is crying). Although systemic side effects are unlikely with the small doses given, we will follow weight and head circumference development as well as glucose levels in blood and urine during the treatment period and at 40 weeks PMA, a time point at which magnetic resonance imaging (MRI) of the brain will also be performed.

2.4.2. Potential Benefits

The benefits of reducing the risk for general anesthesia and destructive laser treatment as well as exposing the infants to anti-VEGF with unknown systemic effects seems substantial. It has in several studies been demonstrated that the poorest outcome both regarding visual and cognitive outcomes are in the infants that have been treated for ROP. It is also well known that infants treated for ROP with laser and/or Anti-VEGF have a higher risk for blindness than untreated.

3. Study Population

Hundred (100) infants screened for ROP are planned to be randomized to either Dexamethasone treatment (n=50) or Placebo (physiologic saline) arm (n=50) at following centers: Region Västra Götaland, Sahlgrenska University Hospital in Gothenburg and Skaraborgs Hospital (SKAS) Skövde, and Region Jönköpings län Ryhov Hospital, and Uppsala Akademiska Hospital and St. Erik Eye Hospital and Karolinska University Hospital Stockholm and Umeå University Hospital.

3.1. Inclusion Criteria

The study will apply following inclusion criteria:

1) Gestational age <30 weeks
2) Zone I ROP without plus disease with either
   a. ROP Stage 1, or ROP Stage 2
   or
   Zone II ROP without plus disease with either
      a. Posterior ROP stage 2
         or
      b. ROP stage 3 with or without notch in zone I
3) ROP stage documented using RetCam photography and confirmed by two ophthalmologists
4) Parent’s informed (oral and written information) consent.

3.2. Exclusion Criteria

The study will apply following exclusion criteria:

1) Ongoing ocular infection
2) If the patient is judged unsuitable for the study by treating neonatologist/paediatrician or ophthalmologist for any other reason

Withdrawal Criteria

The parents and guardians will be informed that they are free to withdraw from the study at any time. A discontinuation occurs when an enrolled infant ceases participation in the study, regardless of the circumstances, prior to completion of the protocol.

The investigator has the right to exclude an infant at any time if it is in the best medical interest of the infant.

Infants will be discontinued from treatment prior to completion for any of the following reasons:

- Consent withdrawn / the parents/guardians wish to discontinue study treatment
- Adverse event (AE, clinical events or laboratory values) that contraindicate continuing the study.
- Best interest of the patient, as judged by the Investigator
- Protocol violation
- Other, by Investigator specified reason

The Study Termination examination / sampling must be performed at the time of the study discontinuation. The investigator will record the reason for study discontinuation, provide or arrange for the appropriate follow-up (if required) for such infant, and document the course of the infant's condition.

3.3. Subject Replacement

Infants who withdraw from the study will not be replaced.

4. Study Schedule

Study schedule including plan and timing of procedures is presented in the table below.

Studie schema:

DROPROP STUDY Schedule									
	**Treatment Phase (Main) Study**							**Extension Study**	
**Procedures**	**ROP screening**	**Clinical visit**	**ROP Screening/ treatment**			**Clinical visit (40w PMA)**	**Termination Visit[b]**	**Follow-up**	
	**Visit R0**	**Visit C1**	**Visit R1**	**Visit R2**	**Visit RX[a]**	**Visit C2**	**Visit T1**	**2.5 yrs Visit F1**	**6.5 yrs Visit F2**
Study Procedures and Data Collection									
Informed consent	X								
Eligibility	X								
Medical history/ demographics infant/mother	X								
ROP evaluations	X		X	X	X				
RetCam photography	X		X	X	X				
Randomization		X							
Dexamethasone/Placebo treatment dispense/return		X	X	X	X	X	X		
ROP treatment with laser and/or anti-VEGF			X[c]	X[c]	X[c]				
Retinal morphology with OCT								X	X
Intraocular pressure	X[d]		X[d]	X[d]			X	X	X
Visual outcome and refractive errors								X	X
MRI[e] + Peapod						X[e]			
Physical examination		X				X		X	X
Weight, Length, Head		X	X	X	X	X		X	X
Vital signs: SPO2, HR, BP		X					X		
Blood samples		X[f]	X[f]	X[f]	X[f]	X[f]	X[f]		
Saliva cortisol		X	X	X	X		X	X (GBG)	X (GBG)
Urine sample		X				X	X		
Adverse events	X	X	X	X	X	X	X		
Diagnoses and complications								X	X
Study termination		X	X	X	X	X	X	X	X

Visit R0= Visit before start of treatment
Visit C1 = Clinical visit 1 (start of treatment)
Visit R1 = 1 week after start of treatment
Visit R2 = 2 weeks after start of treatment
[a]Visit RX = ROP Screening and treatment as many as required in order to be correctly followed-up regarding the monitoring and treatment of the ROP disease, procedures performed weekly.
Visit C2 = Clinical visit 2 (40 weeks PMA)
Visit T1 = 2 weeks after end of treatment
[b] Main study termination visit occurs two weeks after the last treatment or at earliest at 40 weeks PMA when maximum ROP should have occurred.
[c] ROP treatment provided when required according to guidelines.

[d] Intraocular pressure will be measured before treatment, 1 and 2 weeks after the start of treatment, and 2 weeks after the end of treatment, and at follow-up visits.
[e] if performed in clinical routine, PeaPod in Gothenburg
[f] if clinical sample is taken left over blood is collected for analyses

5. Study Design

This is a multicentre, blinded, randomized, controlled study including two parallel arms, Dexamethasone treatment and Placebo (physiologic saline) arm, in 1:1 ratio. Randomisation will stratify for center. Hundred (100) infants will be included at ten centers in Sweden at the time when ROP zone I stage 1 and 2 and ROP stage 2 posterior zone II or stage 3 without plus disease is diagnosed and will be followed up to 40 weeks PMA or end of ROP screening in the study Treatment Phase and in an Extension Follow-up part at 2.5 and 6.5 years of age. Study recruiting process is planned to start in Q4 2022 and is anticipated to last about 2 years.

6. Methods and Assessments

6.1. Randomization and Blinding

Infants eligible for the study will be randomized in strictly sequential order at each center according to the study’s randomization list. They will be randomized to receive eye drops with either Dexamethasone 1 mg/ml (Dexafree®) or physiologic saline provided in similar single-dose containers. Treatment will be of single-blind nature, blinded for the ophthalmologist treating the infants. Randomization will be performed stratified by center using an electronic application. Each randomization number will have a sealed envelope available at the center, stored safely in a locker where the randomized treatment is listed in case of safety issues and need for unblinding among the study personnel.

6.2. Study Visits and Procedures

6.2.1. Treatment Phase Visits and Procedures

Eligibility, Informed Consent and Randomization

Infants evaluated for eligibility regarding inclusion into the study will be identified at the routine ROP screening checks performed following national guidelines. Parents/guardians will be informed about the study and asked to participate if the infant is found eligible. The written informed consent will be collected before any study-specific data is measured and collected, and before the infant is randomized to either Dexamethasone treatment or Placebo arm.

ROP evaluations

ROP evaluations will follow the standardized protocol and adhere to clinical screening praxis according to national guidelines. They will be comprised of as many ROP screening visits as necessary for the monitoring and treatment of the ROP disease. Those visits will be denoted as Visit R0-Visit RX. Information about ROP screening date, stage, zone, location, plus disease and ROP treatment will be collected for both eyes. The evaluation of ROP will occur independently from the study and the pediatric ophthalmologists will be unaware of if the patient receive placebo or active Dexafree® treatment.

RetCam photography

At each ROP screening visit RetCam will be used to document the extent of the ROP disease (central, nasal, temporal, inferior and superior images) and images will be saved in the clinical record file.

Saliva cortisol

Samples for salivary cortisol are taken before treatment and thereafter once a week during treatment and at T1, F1 and F2. In Gothenburg (n = 30), salivary cortisol levels are measured at follow-up visits at 2.5 and 6.5 years.

Medical history/demographics

Infant's and mother's following demography and medical history data will be collected:

- Infant
    - Date of birth
    - GA at birth
    - Sex
    - Birth weight (g)
    - Birth length (cm)
    - Birth head circumference (cm)
    - Medical history
        - Presence of Bronchopulmonary dysplasia (BPD)
        - Presence of Necrotizing enterocolitis (NEC)
        - Presence of Sepsis
- Mother
    - Age (years)
    - Medical history
        - Diabetes
        - Hypertension
        - Other disease requiring medication
    - Pregnancy complications
        - Preeclampsia
        - Infection requiring antibiotics
        - Other disease requiring medication

Dexamethasone/Placebo treatment

Study treatment will be given to the infant in the neonatal department by a study nurse. In cases where the infant is at home a number of single doses for one week will be provided to the parents/guardians for application at home by the parents. Dispensed number of doses will be reported at each visit as required at visits C1, R0-RX and C2, and number of returned single doses at the termination visit T1.

ROP treatment with laser and/or anti-VEGF

The criteria for conventional treatment is completely independent from the trial and ROP treatment will be performed according to the ETROP treatment criteria (Early Treatment for Retinopathy of Prematurity Cooperative Group for all infants included in the study independent of treatment arm. Revised indications for the treatment of retinopathy of prematurity. Arch Ophthalmol 2003;121(12):1684-1696.). If there is a consideration of Anti-VEGF treatment, eye drops should be stopped immediately. After laser treatment, 2-3 drops daily are given for 7 days, then tapering off the dose.

Intraocular pressure

To assess possible adverse effects intraocular pressure will be measured before dexamethasone treatment is given at visit R0 and after 1 and 2 weeks of treatment at visits R1 and R2 and after end of treatment at visit T1. It will also be measured at follow-up visits at 2.5 and 6.5 years.

MRI

MRI of the brain will be performed at 40 weeks PMA (Visit C2) according to clinical routine. MRI will be performed using a 3 Tesla.

Physical examinations

Weight, length and head circumference will be followed during treatment at all clinical study visits (Visit C1 and C2) as well as body composition with PeaPod at 40 weeks in Gothenburg.

Vital signs

Oxygenation (SpO2), heart rate and blood pressure will be measured before the treatment and after (Visit C1 and T1).

Urine and blood samples

Glucose in urine will be checked after initial start of treatment and at 40 weeks PMA. If treatment continuous after 40 weeks PMA, a urine sample will be taken 2 weeks after completed treatment, at the main study termination visit T1.

Blood samples left over from clinical sampling will be analysed with regard to factors involved in ROP related processes such as angiogenesis, neurogenesis, inflammation and others.

Adverse events

Adverse events will be reported throughout the study. For detailed definitions and reporting guidelines see chapter 9 below.

6.2.2. Treatment Phase Study Termination Visit

The treatment phase (main) study termination visit is planned to occur at PMA 40 weeks or two weeks after last ROP treatment given (Visit T1). The end of the trial is defined as the last visit of the last subject included in the trial.
For infants who early discontinue from the study, the study termination examination/sampling must be performed at the time of the study discontinuation instead. The investigator will record the reason for study discontinuation, provide or arrange for the appropriate follow-up (if required) for the infant, and document the course of the infant's condition.

This study may prematurely terminate for all enrolled infants for any of the following reasons:

- It was determined that the risk level associated with the investigational medication was significant and warranted termination of the study;
- The sponsor terminated the study for any reason including following a recommendation from the Data Monitoring Committee (DMC), at any time, by written notice of intended termination;
- The principal investigator, EPN terminated participation of a specific clinical center in the study by written notice;
- Any other clause in the individual center Clinical Study Agreement was not met.

6.2.3. Extension Study Follow-up and Procedures

Infants will be followed up according to National Guidelines as an extension part of the study, post unblinding and post main study data publication, when they reach 2.5 and 6.5 years of age.

Following data will be collected according to the same procedures that were used during the treatment phase (main) study:

- Retinal morphology with OCT
- Intraocular pressure
- Visual outcome and refractive errors
- Physical examinations

- Vital signs
- Blood samples
- Salivary cortisol sample (Gothenburg n=30)

Additionally, severe diagnoses and complications will be collected, including but not limited to the following ones:

- Presence of Glaucoma

Retinal morphology with OCT

OCT will be performed at 2.5 and 6.5 years evaluating both the optic disc and maculae region.

Visual outcome and refractive errors

Examination at 2.5 years will include (visit F1);
1) Best-corrected vision assessed binocularly and monocularly, if possible. Three different tests with gradually decreasing difficulty will be used: 1. able to identify single optotypes 0.4 LH (Lea Hyvärinen) test at 3 m distance, 2. able to fixate and follow a toy of 5 cm at 30 cm, 3. able to fixate and follow a light/torch at 30 cm. Children/eyes who will not be able to identify an optotype at 3 m or a toy at 30 cm, will be regarded to have impaired vision. Children/eyes who will not be able to fixate and follow a light will be regarded to be blind.
2) Ocular motility and nystagmus will be assessed and covertest will be performed for near (30 cm). 3) Strabismus will be defined as "manifest strabismus", i.e. esotropia, exotropia or hyper/hypotropia. Evaluation of stereopsis will be performed with Lang test.
4) Refraction will be assessed with retinoscopy in cycloplegia. Automatic refraction (Retinomax) in cycloplegia will be performed if retinoscopy is not possible.

Examination at 6.5 years will include (Visit F2);
1) Best-corrected vision will be assessed binocularly and monocularly with KM test if not possible to perform HVOT test will be performed.
2) Ocular motility and nystagmus will be assessed and covertest will be performed for near (30 cm).
3) Strabismus will be defined as "manifest strabismus", i.e. esotropia, exotropia or hyper/hypotropia. Evaluation of stereopsis will be performed with Lang test.
4) Intraoccular pressure will be performed
5) Refraction will be assessed with retinoscopy in cycloplegia. Automatic refraction in cycloplegia will be performed if retinoscopy is not possible.

7. Trial Materials

7.1. Trial Products

Study patients will be randomized to receive eye drops with either Dexamethasone 1 mg/ml (Dexafree®) or physiologic saline.

Dexafree®

Thea Nordic has received marketing authorization for Dexafree® eyedrops 1 mg/ml for ocular treatment.

Physiologic saline

Drop-it is a steril, buffered physiologic saline without preservative with the same content of sodium chloride as the tear film.

7.2. Storage and Accountability

The local hospital at each study center will be responsible for storing and dispensing all supplies. The Dexafree® and the physiologic saline will be stored at room temperature until patient is included in the study and the product administered.

8. Study Treatment

8.1. Rationale for Selection of Dose

The rationale for dose selection is based on one years of Dexafree® use at the pediatric ophthalmology department in Lund, Skåne where infants with moderately severe ROP have been treated with Dexafree® to prevent severe ROP needing treatment. The dose of Dexafree® has been titrated out to be efficient and still give a minimum dose of the medication. During the last year only one infant in Lund has developed severe ROP needing treatment. Historically 7-10 infants/year have needed treatment for severe ROP in Lund.

8.2. Study Treatment Formulation

The investigational products Dexafree® and the physiologic saline have the eye drop formulation.

8.3. Study Treatment Administration

When an infant is included in the study, randomized to receive eye drops either with Dexafree® or the physiologic saline and assigned a randomization number by the neonatology nurse using an electronic application, the treatment will be administered at the hospital by the neonatology nurse when the infant is admitted to neonatal care. The screening ophthalmologist will not be aware of the randomized treatment. Singe doses will be dispensed to the parents for one weeks use if the child is in home care and information provided about proper administration. The parents will record given doses in a digital App. The total number of single doses used and compliance will be estimated by

collecting information about returned number of doses. It will be emphasized that it is important to have all unused doses of investigational product returned back to the center.

The eye drops will be given at a dose of 1 drop every second day to one drop daily in affected eyes. If ROP regresses the dose will be lowered to 1 drop every second day If ROP worsens when 1 drop every second day is given increase drops till once daily. Continue dropping in infants with ROP type 2, one drop daily or one drop every second day (see below) until there is no type of ROP present. If the infant has had one drop every second day stop treatment. If the infant has had one drop daily, then drop once every second day for one week and the stop treatment. If ROP worsens drop according to schedule below. If drop treatment is finalised, new screening control after a week.

Maximal treatment duration will be 12 weeks.

If Type 1 ROP develops the dose will be increased to 1 x 3 daily before treatment and after treatment 2 drops daily are given for 5 days, then tapering off the dose with 1 drop for two days.

	**Number of drops/dag**
Zone 1, ROP stage 1-2 without plus	1 drop every second day
Zone 2, **posterior zone 2**, ROP stage 2 without plus	1 drop every second day
Zone 2, ROP stage 3, with or without notch in zone 1, without plus	1 drop every day
Type 1 ROP	1x3 drop until treatment
Treatment and post treatment	routines at the clinic

8.4. Specific Restrictions/Requirements

After opening the protective bag containing the single-dose containers, the single-dose containers should be used within 15 days. The single dose containers should be used immediately after opening and discarded after use. The single-dose containers should be stored in their original packaging. They are sensitive to light.

8.5. Blinding

Thea Nordic will supply Dexafree® in the study in single-dose containers.

Physiologic saline will be supplied in single-dose containers ("Drop it") similar to those for Dexafree®. Identification of drop content will not be visible for an untrained eye. The neonatology nurse, dedicated for randomization, administration of the first dose of the treatment and distribution of the doses to parents/guardians. Sealed envelopes will information about treatment arm corresponding to each randomization number will be safely stored in a locker at the respective center, available to be unsealed in case of emergency and safety issues at investigator's initiative. At the end of the study, the monitor will confirm that no envelopes other than the known unblinded ones were opened during the study.

Unblinding is planned to be done when the treatment phase (main) study has reached completion, the study database has been monitored, reviewed, cleaned and locked.

8.6. Concomitant Therapy

Eyes progressing to treatment warranting ROP (Type 1) according to international standards (2) will receive conventional laser and/or possibly anti-VEGF treatment within 72 hours from detection.

9. Safety Measurements

9.1. Definitions

9.1.1. Adverse Events

Clinically significant changes (abnormalities), in the judgment of the investigator, from the physical examination of the infant will be recorded as adverse events starting from the time of randomization (visit C1) until the treatment phase termination visit (visit T1). Any medical condition present at the initial study day (the date of informed consent), which remains unchanged or improves, should not be recorded as an adverse event at subsequent examination / sampling days. However, if there is deterioration of a medical condition that was present at the initial study day (informed consent), this should be considered a new adverse event and reported. Ongoing adverse events at the treatment phase termination visit will be followed up until the event is resolved or remains stable.

Especially 1) increased ocular pressure (>20 mmHg) 2) abnormal growth (>1SD diff in weight, length and head circumference deviation) after initiation of dexamethasone drops.

9.1.2. Serious Adverse Events

A serious adverse event (SAE) related to the investigational product will be defined as any adverse medication experience occurring at any dose that results in any of the following outcomes:

- Results in death,
- Is life-threatening (NOTE: The term "life-threatening" in the definition of "serious" refers to an event in which the infant was at risk of death at the time of the event; it does not refer to an event which hypothetically might have caused death if it was more severe).

- Results in inpatient hospitalization or prolongation of existing hospitalization,
- Results in a persistent or significant disability/incapacity, or
- Results in a congenital anomaly/birth defect.

Important medical events that may not result in death, be life-threatening, or require hospitalization may be considered a serious adverse medication experience when, based upon appropriate medical judgment, the event may jeopardize the infant and may require medical or surgical intervention to prevent one of the outcomes listed in this definition.

Suspected unexpected serious adverse reaction (SUSAR). A SUSAR is every suspected adverse reaction which is both unexpected and serious.

In case of a SUSAR the Coordinating Investigator together with the Sponsor is responsible to inform – by submitting an expedited report CIOMS-blankett (se Appendix 1) a.s.a.p. to registrator@mpa.se.

The time limits are: 7 days in case of death or life-threatening SUSAR, and 15 days otherwise.

The Sponsor is responsible for informing all investigators in Sweden according to local legal requirements. They are also responsible for providing any support in SUSAR or other safety submissions to their national competent authority or Ethics Committee according to local requirements.

9.2. Collecting, Recording and Reporting Adverse Events

9.2.1. Adverse Events

The following information must be collected and recorded for each AE:

- AE term (diagnosis)
- AE start and end date
- Action taken regarding AE
- AE outcome (resolved, ongoing, death, or lost to follow-up)
- AE causality (not related, possibly related, related)

The investigator should determine relationship to the investigational product for each adverse event. AEs occurring prior to administration of the study medication will be considered as not related to the study medication. The Investigator will be using the following explanations for assessment of causality:

Not related

The event is clearly related to other factors such as the infant's clinical state, therapeutic interventions, or concomitant medications administered to the infant.

Possibly Related

The event follows a reasonable temporal sequence from the time of medication administration,
And/or follows a known response pattern to the study medication.
But could have been produced by other factors such as the infant's clinical state, therapeutic interventions, or concomitant medications administered to the infant.

Related

The event follows a reasonable temporal sequence from the time of medication administration, And follows a known response pattern to the study medication, And cannot be reasonably explained by other factors such as the infant's clinical state, therapeutic interventions, or concomitant medications administered to the infant. And either occur immediately following study medication administration, or improves on stopping the medication, or reappears on repeat exposure, or there is a positive reaction at the application center.

It is up to the discretion of the Investigator, in the event of an AE (or SAE), to temporarily discontinue study medication or to discontinue the infant from the study. The study termination examinations / samplings must be performed if the infant is discontinued from the study.

9.2.2. Serious Adverse Events

Any SAE to study drug occurring during the study (from randomization until end of study) will be reported from the PI at each site to the Sponsor within 24 hours of knowledge of the event.

In the event of an SAE, the SAE form available in the electronic Trial Master File (TMF) is to be completed and reported to Ulf Westerlund, Nordic Marketing manager within 24 hours of learning about the event regardless of whether all information is known. An e-mail will also be sent to ulf.westerlund@theapharma.com to inform about the SAE.

Initial SAE reporting can be done by telephone with written reports to follow by fax to the sponsor within 24 hours.

All SAEs must be followed until resolution (infant has returned to baseline status of health), or until stabilization (the investigator does not expect any further improvement or worsening of the reported event). Also, infants who withdraw from the study for an SAE should be followed until the SAE has resolved. Appropriate supportive and/or definitive therapy should be administered as required.

A discontinuation due to an AE/SAE must be reported immediately to Thea Nordic by the sponsor.

Sponsor Safety Contact Information

Contact: Ann Hellström
Title: MD Professor
Address: The Queen Silvia Children's Hospital,
Göteborg University/Östra
SE – 416 85 GÖTEBORG

24 hour line: +46 (0)768 979196
E-mail: ann.hellstrom@medfak.gu.se
Office: +46 (0)31 3435774
Fax: +46 (0)31 3435771

Safety Contact Information for Thea Nordic:

Contact: Eric Iwar, Théa Nordic, Örebro, Sweden
Title: Nordic Marketing Manager
Address: Storgatan 55, 703 63 Örebro, Sweden
E-mail: eric.iwar@theapharma.com
Tel: +46 705154632

9.3. Safety Monitoring Plan

Before, during and after the study, the monitor will have regular contacts with the clinic including visits to confirm that facilities remain acceptable, that the investigational team is adhering to the protocol, that data are being accurately recorded in the CRF and to provide information and support to the Investigator. Monitoring of the study will be carried out by clinical research manager Carola Pfeiffer Mosesson, Institute of Neuroscience and Physiology, University of Gothenburg according to their Standard Operating Procedures (SOP).

The investigator agrees to facilitate the monitoring of the study by a Sponsor representative and Regulatory Authorities to have the right from time to time both during and after the course of this study to inspect the study procedures and review pertinent medical records.

9.4. Training

The Investigator will ensure that appropriate Good clinical practice training relevant to the study is given to the medical, nursing and other staff involved. Any information of relevance to the performance of this study is to be forwarded to the co-investigators and other staff involved.

All primary investigators (PI) signing the protocol and key personnel should provide signed and dated Curriculum Vitae (CV) originals to be filed by the Sponsor. The CV should include name, title, occupation, education, research experience and present and former positions. A staff signature list including delegation responsibilities is required and will be continuously updated.

10. Data Management

10.1. eCase Report Forms

The PI will be responsible for the database containing the information collected in the study retrieved from the eCase Report Forms (eCRFs). The eCRFs will be developed using HOPE platform from ADDI Medical (www.addimedical.se)

Completed original eCRF are the property of the Sponsor.

10.2. Data Quality Assurance

The data will be entered into a database, where internal review and programmed computer checks will be used to identify selected protocol violations and data errors. If necessary, requests for clarifications or corrections will be sent to the investigator.

A statement will be obtained from each infant's parents/guardians participating in the trial permitting the release of the infant's medical records as necessary for monitoring or inspection by authorized personnel for the Sponsor and Regulatory Authorities.

The investigator is responsible for maintaining a comprehensive and centralized filing system of all study-related (essential) documentation, suitable for inspection at any time by representatives of the Sponsor and Regulatory Authorities.

The investigator will sign-off and put a date stamp at the indicated places on the CRFs. These signatures will signify that the investigator inspected or reviewed the data on the CRF and on the data queries and that he/she agrees with the content.

All corrections on an eCRF and on source documents must be made in a way that does not obscure the original entry. Any changes in the eCRF will be logged in the system Corrections necessary after the CRFs have been completed must be documented on a Data Clarification Form (DCF).

10.3. Data Entry and Storage

Original source documents, eCRFs, and other study documentation will be maintained at the study centers. Source data will be collected in the "Source Data File". The Source Data File should contain raw data from examinations/samplings/laboratory results required to verify the data entered into each infant's eCRF. All original source documents supporting entries on eCRFs must be maintained and be readily available at the center.

The investigator will record all data with respect to the study in the infant's eCRFs. Data will be stored at Gothenburg University Hospital.

11. Sample Size and Statistical Methods

11.1. Determination of Sample Size

Natural history of ROP has shown that 50% of the infants with stage 2, zone II ROP with hemorrhage or stage 3, zone II develop Type 1 ROP needing treatment (1) . We hope to reduce the number of infants needing treatment with laser and/or Anti-VEGF by 60%. Assuming 50% infants needing ROP treatment in the Placebo arm and 20% in the Dexamethasone treated arm, alpha 0.05, using two-sided Fisher's exact test, would require 45 infants to be included per study arm (1:1 ratio), in total 90 infants excluding drop-outs, in order to achieve a power of 80%. Taking into account 10% drop-outs, 50 infants per study arm, 100 infants in total will be needed to be included.

Approximately 45 infants/year develop severe ROP in Region Västra Götaland (Sahlgrenska University Hospital and Skaraborgs Hospital), Stockholm Region, Umeå Region, Region Jönköping (Ryhovs Hospital). Hence, the study's enrolment period is estimated to last just over two years.

11.2. Statistical and Analytical Plan

11.2.1. Analysis Populations

Intention-to-treat population

Intention-to-treat (ITT) population will include all randomized infants correctly included in the study and will be analysed on as-randomized basis. Infants wrongly included in the study will be excluded from the ITT population, but will be part of the safety population.

Per protocol population

Per protocol (PP) population will include all infants that are part of the ITT population that do not have any significant protocol violations. Protocol violations will be continuously collected in the study. The reasons for exclusion from the PP population will be reviewed and finalized during the clean file meeting before the database lock. Per protocol analysis will be performed on as-treated basis.

Safety population

All infants included in the study that have received at least one dose of either of the two study arms will be part of the safety population. Safety analysis will be performed on as-treated basis.

11.2.2. General Considerations

All detailed statistical methods will be documented in a Statistical Analysis Plan (SAP) for the study. The SAP will be finalized as soon as possible after the finalization of the study protocol and eCRFs.

All analyses will be performed using either SAS software version 9.4 or later (SAS Institute Inc, Cary, NC, USA) or SPSS software version 26.0 or later (IBM Corp, Armonk, NY).

For descriptive purpose mean, standard deviation (SD), median, minimum and maximum will be presented for continuous variables and number and percentages for categorical variables.

For test between the two study arms regarding infant's characteristics at study start, Fisher's exact test will be used for dichotomous variables, Mantel-Haenszel Chi-square trend test for ordered categorical variables, Chi-square test for non-ordered categorical variables and Fisher's non-parametric permutation test for continuous variables.

All efficacy evaluations will be adjusted for center (used as strata in randomization) and gestational age (the most prominent risk ractor).

Evaluation of difference between the two study arms with respect to binary outcomes will be performed using logistic regression, and graphically presented by bar charts. Missing data for early

discontinuations will be considered as no event. Sensitivity analysis will study the missing values considered being event instead.

Evaluation of time to event data between the two study arms will be performed using Cox proportional hazards model, and graphically presented by Kaplan-Meier graphs. Missing data will be handled through censoring.

Evaluation of number of recurrences per follow-up time between the two study arms will be described by event rates (number of events divided by the number of follow-up days) and will be compared using Poisson regression.

Evaluation of difference between the two study arms with respect to continuous outcomes will be performed using analysis of covariance (ANCOVA) if analyses assumptions might be shown fulfilled, otherwise, unadjusted analyses will be performed using Fisher's non-parametric permutation test, and graphically presented by scatter plots or boxplots.

Evaluation of difference between the two study arms with respect to continuous outcomes repeatedly measured over time will be performed using mixed models for repeated measures (MMRM) if analyses assumptions might be shown fulfilled adjusted for before treatment value (beside center and GA), otherwise, unadjusted analyses will be performed using Fisher's non-parametric permutation test, and graphically presented by scatter plots or boxplots.

All efficacy variables will be evaluated on the ITT and PP populations, ITT being the main analysis. All safety variables will be evaluated on the safety population.

All tests will be two-tailed.

The primary analysis will be considered confirmed if $p<0.05$.

We are aware of the information concerning the recommended management of clinical trials during the Covid-19 pandemic in the guidance document agreed on by the Clinical Trials Expert Group (CTEG) of the European Commission supported by the EMA, the Clinical Trials Facilitation and Coordination Group (CTFG) of the Heads of Medicines Agencies (HMA) and the GCP Inspectors' Working Group coordinated by the EMA. And will continuously update on https://ec.europa.eu/health/sites/health/files/files/eudralex/vol-10/guidanceclinicaltrials_covid19_en.pdf

and on EMA website: https://www.ema.europa.eu/en/implications-coronavirus-disease-covid-19-methodological-aspects-ongoing-clinical-trials

11.2.3. Primary Efficacy Analysis

The primary outcome, the proportion of infants with ROP progression to Type 1 ROP needing conventional treatment, will be evaluated for difference between the two treatment groups using logistic regression, adjusted for the center used as stratifying variable in the randomization procedure and GA that is the most prominent predictor for Type 1 ROP. In this analysis, missing data for early discontinuations will be handled as *non-events*. Odds-ratio (OR) with 95% confidence intervals (CIs) will be presented along with its p-value. If $p<0.05$ for the analysis performed on ITT population on as randomized basis, the primary analysis will be considered confirmed.

Analysis on PP population on as treated basis will be performed as complementary analysis.

Analysis on ITT population imputing *event* to missing data will be performed as sensitivity analysis.

Graphically, the proportion of infants with Type 1 ROP in the two treatment groups will be described by bar-charts.

Descriptive data will be provided separately per each center. Any center differences will be reported and discussed.

11.2.4. Secondary Efficacy Analyses

The secondary efficacy analyses performed on the secondary outcomes described in section 2.3 will be evaluated according to the methods described in the general methodology above. These analyses will not be included in the confirmatory tests, but described with appropriate estimates and 95% CI. Analyses adjusted for center and GA will be made.

The main analysis will be performed on the ITT population on as randomized basis.

The complementary analysis will be performed on the PP population on as treated basis.

11.2.5. Safety Analyses

The safety data will only be summarized for the two treatment arms on the safety population on as treated basis. No formal statistical tests will be performed.

11.2.6. Data Monitoring Committee

A DMC will be appointed in this study including an independent group of experts in the area that are not the Study Sponsor or part of the study's Steering Committee; Gunnar Jakobsson MD PhD (voting), ophthalmologist, Boubou Hallberg MD PhD, (voting), neonatologist, and Prof Max Petzold (voting), statistician, will scrutinize the data according to below prepared by a non-voting statistical programmer.

Two planned closed DMC meetings will be held, the first one when 35% of the infants have been followed up until the main study end, and the second one when 70% of the infants have been followed up until the main study end. At both time points DMC will analyse data for efficacy (benefit) and for safety (harm). Ó'Brian-Flemming group sequential boundaries on positive side will be applied for evaluation of potential stopping for benefit, and z-value exceeding 2.40 will be applied for evaluation of potential stopping for harm. DMC may, if found necessary, schedule additional DMC meetings in-between planned closed meetings.

Information about study results will be strictly held within the closed DMC group. After a closed DMC meeting, the Sponsor and the study's Steering Committee will only receive a recommendation to

Stop or *Continue* the study. The decision on any actualization will be made by the study's Steering Committee.

A detailed plan describing the DMC's work will be available in a DMC charter, written and signed off before the start of the first DMC analyses.

12. Ethical Considerations

12.1. Independent Ethics Committee

The protocol, informed consent form, and other written infant information will be submitted to the "Etikprövningsmyndigheten" (EPN, www.epn.se) and their written unconditional approval must be obtained prior to commencement of the study.

Verification of unconditional approval from the EPN of the protocol and the approved informed consent form will be forwarded to Thea Nordic, the manufacturer of the study medication, prior to shipment of study medication supplies to the center.

12.2. Informed Consent Form

Informed consent by the parents/guardians for each infant will be obtained before initiating any study procedures. Both parents/guardians will have to sign the informed consent. One copy of the signed and personally dated informed consent must be given to each parent/guardian and one signed and personally dated copy must be retained in the investigator's trial records. The "Declaration of Helsinki" recommends that consent be obtained from each potential infant or parents/guardians in biomedical research trials after the physician has explained to the individual the purpose, methods, anticipated benefits, and potential hazards of the trial and discomfort it may entail.

Potential infant's parents/guardians should also be informed of their right not to participate or to withdraw from the study at any time. If the individual is in a dependent relationship to the physician or gives consent under duress, an independent physician should obtain the informed consent. If the individual is legally incompetent (i.e., a minor or mentally incompetent), informed consent must be obtained from the parents, legal guardians, or legal representative in accordance with the law in Sweden. See the "Declaration of Helsinki".

If a protocol amendment substantially alters the study design or there is an increase of a potential risk to the infant:

- The informed consent form or infant information sheet must be revised and submitted to EPN for review and approval; and

- The approved revised form must be signed by parents/guardians to infant currently enrolled in the study; or
- The new form must be used to obtain consent from new parents/guardians prior to enrollment into the study.

12.3. Confidentiality of Data and Patient Records

The Investigator is responsible for keeping a list of all randomized infants including randomization numbers, full names of parents/guardians and child and parents addresses.

The parents/guardians should also be informed in writing and agree to the possibility of audits and/or monitoring by authorized representatives of the Sponsor, the manufacturer of the study medication, and/or regulatory authorities in which case a review of those parts of the laboratory records relevant to the study may be required.

The parents/guardians should be informed in writing and agree to that the results will be stored and analyzed in a computer, maintaining confidentiality in accordance with the Swedish Personal Data Ordinance (1998:1191). "Personuppgiftslagen" (PUL SFS 1998:204, SFS 1998:1191)

12.4. Biobank

Samples taken in this study will be stored in a biobank according to the National Swedish Board of Health and Welfare in accordance with the Biobanks in Medical Care Act (2002:297). The biobank is registered at the national board of health and welfare in Sweden (Socialstyrelsen). The parents/guardians will be informed about this.

13. Publications

Two main articles are planned to be produced, the first when the treatment phase is finalized at around 40 weeks of PMA, and the second when the extension part is finalized, including safety follow-up visits at 2 and 5.5 years. The articles will be written and published by the study investigators.

Additionally, following sub-studies are planned to be evaluated from the study database and published:

- Center differences in ROP development and treatment
- Biomarkers in relation to Dexafree® treatment and ROP development

14. Retention of Trial Documents

14.1. Study Data

ICH-GCP guidelines require the medical records and notes, etc., should be clearly marked and permit easy identification of participation by an individual in the trial.

The files will be archived by the investigator at least 10 years after the study is finished and the study records will be stored in a secure server.

14.2. Protocol Amendments

All revisions to the protocol should be reviewed and approved by the Sponsor prior to submission of the amendment to the regulatory authorities. If the revision is an administrative change, the investigator should submit it to the EPN for their information. If the revision is an Amendment to the protocol, the Principal Investigator must sign off that version to verify he/she has read and understands the change. The investigator must submit the Amendment to the EPN for review and approval prior to implementation.

If an amendment substantially alters the study design or increases the potential risk to the infants:

- The informed consent form or patient information sheet must be revised and submitted to the EPN for review and approval; and
- The approved revised form must be signed by both parents/guardians currently enrolled in the study; and
- The new form must be used to obtain consent from new parents/guardians prior to their enrolment into the study.

15. References

1. Holmstrom G, Hellstrom A, Granse L, Saric M, Sunnqvist B, Wallin A, et al. New modifications of Swedish ROP guidelines based on 10-year data from the SWEDROP register. Br J Ophthalmol. 2019.
2. group Etfropc. Revised indications for the treatment of retinopathy of prematurity. Arch Ophthalmol. 2003;121:1684.
3. International Committee for the Classification of Retinopathy of P. The International Classification of Retinopathy of Prematurity revisited. Arch Ophthalmol. 2005;123(7):991-9.
4. Bengani LC, Kobashi H, Ross AE, Zhai H, Salvador-Culla B, Tulsan R, et al. Steroid-eluting contact lenses for corneal and intraocular inflammation. Acta Biomater. 2020.
5. Iovino C, Mastropasqua R, Lupidi M, Bacherini D, Pellegrini M, Bernabei F, et al. Intravitreal Dexamethasone Implant as a Sustained Release Drug Delivery Device for the Treatment of Ocular Diseases: A Comprehensive Review of the Literature. Pharmaceutics. 2020;12(8).
6. Doyle LW, Cheong JL, Ehrenkranz RA, Halliday HL. Early (< 8 days) systemic postnatal corticosteroids for prevention of bronchopulmonary dysplasia in preterm infants. Cochrane Database Syst Rev. 2017;10:CD001146.
7. Doyle LW, Cheong JL, Ehrenkranz RA, Halliday HL. Late (> 7 days) systemic postnatal corticosteroids for prevention of bronchopulmonary dysplasia in preterm infants. Cochrane Database Syst Rev. 2017;10:CD001145.
8. Walsh BH, Paul RA, Inder TE, Shimony JS, Smyser CD, Rogers CE. Surgery requiring general anesthesia in preterm infants is associated with altered brain volumes at term equivalent age and neurodevelopmental impairment. Pediatr Res. 2020.
9. Sankar MJ, Sankar J, Chandra P. Anti-vascular endothelial growth factor (VEGF) drugs for treatment of retinopathy of prematurity. Cochrane Database Syst Rev. 2018;1:CD009734.

Sponsor and Principal Investigator

I am responsible for ensuring that this protocol contains all the essential elements for conducting the study. I will provide the protocol and all other important study-related information to the responsible examiners so that they can complete the study in the right way. I am aware of my responsibility to keep the staff working on the study informed and educated.

______________________________ ______________________________

Signatur Date and Place

____

Name Clarification
