# Supplementary material for: Dexamethasone Eye Drops to Prevent Treatment-Requiring Retinopathy of Prematurity: The DROPROP Randomized Clinical Trial
Source: JAMA Pediatr. 2026 Aug 3:e263247. Online ahead of print. doi: 10.1001/jamapediatrics.2026.3247 (PMC13434969; doi:10.1001/jamapediatrics.2026.3247)
Supplement: Supplementary file 2 [file jamapediatr-e263247-s002.pdf]

APNC
Statistics in Life Science

Statistical Analysis Plan
Final

A Randomized Intervention, Multi-Center Study to Determine the Role of Dexamethasone eye drops against proliferative retinopathy of prematurity

March 11, 2024

Authored by (electronic signature):
Aldina Pivodic, Study Statistician

Approved by (electronic signature):
Ann Hellström, Principal Investigator

Revisions:

Nr	Description	Date

Signed document (nngzKR)

APNC

Statistics in Life Science

Table of Contents

1 Background ... 5

2 Study Objectives and Hypothesis ... 5

2.1 Primary Objective and Hypothesis ... 5

2.2 Secondary Objectives ... 5

3 Study Design ... 6

3.1 Treatment Groups/Exposure ... 6

3.2 Flow Chart ... 7

4 Study Populations ... 8

4.1 Intention-to-Treat Population ... 8

4.2 Per Protocol Population ... 8

4.3 Safety Population ... 8

5 Study Variables ... 8

5.1 Baseline Characteristics Variables ... 8

5.2 Medical History ... 9

5.3 Pregnancy Data, Complications and Medications ... 10

5.4 Postnatal Medications ... 10

5.5 Efficacy/Outcome Variables ... 10

5.5.1 Primary Variable ... 10

5.5.2 Secondary Variables ... 10

5.5.3 Exploratory Variables ... 11

5.6 Safety Variables ... 11

5.6.1 Exposure and Compliance ... 11

5.6.2 Adverse Events ... 11

5.6.3 Laboratory Data ... 11

5.6.4 Retinal Examination ... 12

5.6.5 Intraocular Pressure ... 12

5.6.6 Brain Magnetic Resonance Imaging ... 12

5.6.7 Body Composition ... 12

5.6.8 Vital Signs ... 12

6 Statistical Methods ... 12

6.1 Sample Size ... 12

6.2 General Methodology ... 12

Signed document (nngzKR)

6.3 Adjustment for Type I Error 13
6.4 Handling of Missing Data 13
6.5 Confounders/Covariates 14
7 Statistical Analyses 14
7.1 Patient Disposition and Datasets Analysed 14
7.2 Protocol Deviations 14
7.3 Baseline Characteristics 14
7.4 Medical History 14
7.5 Pregnancy Data, Complications and Medications 14
7.6 Postnatal Medications 14
7.7 Efficacy Analyses 14
7.7.1 Primary Efficacy Analysis 14
7.7.2 Secondary Efficacy Analyses 15
7.7.3 Exploratory Efficacy Analyses 15
7.7.4 Subgroup Analyses 15
7.8 Safety Analyses 15
7.8.1 Exposure and Compliance 15
7.8.2 Adverse Events 15
7.8.3 Laboratory Data 16
7.8.4 Retinal Examination 16
7.8.5 Intraocular Pressure 16
7.8.6 Brain Magnetic Resonance Imaging 16
7.8.7 Body Composition 16
7.8.8 Vital Signs 16
8 Interim Analyses 16
9 Changes from Study Protocol 17
10 Planned Tables and Figures 17

Signed document (nngzKR)

Abbreviations

**AE**	Adverse event
**ANCOVA**	Analysis of covariance
**APROP**	Acute posterior retinopathy of prematurity
**CI**	Confidence interval
**DMC**	Data monitoring committee
**GA**	Gestational age
**HELLP**	Hemolysis, elevated liver enzymes, and low platelet count
**IOP**	Intraocular pressure
**ITT**	Intention-to-treat
**IVH**	Intraventricular hemorrhage
**MRI**	Magnetic resonance imaging
**NEC**	Necrotizing enterocolitis
**OCT**	optical coherence tomography
**PDA**	Patent ductus arteriosus
**PMA**	Postmenstrual age
**PNA**	Postnatal age
**PP**	Per protocol
**PT**	Preferred term
**ROP**	Retinopathy of prematurity
**SAE**	Serious adverse events
**SOC**	System organ class
**VEGF**	Vascular endothelial growth factor

Signed document (nngzKR)

1 Background

Retinopathy of prematurity (ROP) affects primarily extremely preterm infants and is characterised by a first phase of arrested retinal vascularization after birth. In severe cases ischemia and inflammation in the peripheral avascular retina cause vascular endothelial growth factor (VEGF) induced pathologic neovascularisation and, in the worst case, retinal detachment and blindness. To prevent blindness approximately 40-50 infants per year are treated with retinal laser photocoagulation in general anaesthesia or in a few cases with intravitreal anti-VEGF therapy. Indications for treatment are regulated by international guidelines based on an international classification of ROP. When treatment warranting ROP has been diagnosed, treatment should be performed within 72 hours. A minor portion of infants are treated for central ROP (zone I). These cases carry the greatest risk of rapid deterioration and so called acute posterior ROP (APROP). In the majority of infants, treatment is performed in the middle zone (zone II) while indications for treatment are lacking for zone III disease.

Dexamethasone is a synthetic glucocorticoid commonly used on the ocular surface or intraocular to treat inflammation and neovascularisation. Intravenous or oral dexamethasone is used to wean preterm infants from the ventilator. Used to prevent bronchopulmonary dysplasia an initial high and thereafter tapering dose 0.5mg/kg per day of dexamethasone the first day of life resulted in increased risk of cerebral palsy and impaired motor and cognitive development. However, treatment after one week of age and lower doses have not been shown to have the same adverse effects.

Of infants with neovascular ROP approximately 50% will eventually need treatment with laser and/or anti-VEGF. If treatment with dexamethasone eye drops is efficient in preventing progression to treatment warranting ROP, infants may escape general anaesthesia. Surgery in general anaesthesia in preterm infants is associated with reduced cerebral white matter volumes and worse neurodevelopmental outcome at two years of age. Retinal laser therapy destroys large parts of the peripheral retina and after intravitreal injection, anti-VEGF agents enter the blood stream where they suppress VEGF with unknown long term effects.

2 Study Objectives and Hypothesis

2.1 Primary Objective and Hypothesis

The primary objective in this study is to examine whether preventative treatment with topical Dexamethasone treatment may reduce the proportion of infants who need conventional ROP treatment in comparison to Placebo arm.

The primary objective will be evaluated on the primary outcome that is:

- The proportion of infants with ROP progression to Type 1 ROP needing conventional treatment in each study arm.

The hypothesis is that proportion of infants with ROP progression to Type 1 ROP needing conventional treatment is lower in the Dexamethasone arm compared to the Placebo arm.

2.2 Secondary Objectives

The secondary objectives are:

Signed document (nngzKR)

- To study time from detection of proliferative ROP to Type 1 ROP in Dexamethasone treatment and Placebo arm
- To study recurrences after laser/Anti-VEGF treatment in Dexamethasone treatment and Placebo arm
- To study retinal morphology at 2 and 5.5 years with optical coherence tomography (OCT) in Dexamethasone treatment and Placebo arm
- To study measurements of intraocular pressure before treatment at 1 and 2 weeks after start of treatment and 2 weeks after completed treatment
- To study visual outcome and refractive errors at 2 and 5.5 years of age in Dexamethasone treatment and Placebo arm
- To evaluate safety and tolerability of the treatment through adverse events, clinical chemistry, retinal exam, intraocular pressure, physical examination, MRI of the brain (if clinically performed), body composition with PeaPod (Gothenburg) and vital signs.
- To measure steroids and other biomarkers related to intervention in buccal swabs taken before start of treatment and thereafter weekly until stop of treatment.
- To analyse leftovers of blood from clinical blood sampling with regard to selected biomarkers and their relation to ROP development and treatment effect (EPITOP-Gothenburg site).

3 Study Design

This is a multicentre, blinded, randomized, controlled study including two parallel arms, Dexamethasone treatment and Placebo (physiologic saline) arm, in 1:1 ratio. Randomisation will be stratified for center. Hundred (100) infants will be included at 12 centers in Sweden at the time when ROP zone I stage 1 and 2 and ROP stage 2 posterior zone II or ROP stage 3 zone 2 without plus disease is diagnosed and will be followed up to 40 weeks postmenstrual age (PMA) or end of ROP screening in the study Treatment Phase and in an Extension Follow-up part at 2 and 5.5 years of age. Study recruiting process is planned to start in Q4 2022 and is anticipated to last about 3 years.

Hundred (100) infants screened for ROP are planned to be randomized to either Dexamethasone treatment (n=50) or Placebo (physiologic saline) arm (n=50) at following centers: Region Västra Götaland, Sahlgrenska University Hospital in Gothenburg and Skaraborgs Hospital (SKAS) Skövde, and Region Jönköpings län Ryhov Hospital, and Region Uppsala, Uppsala Akademiska Hospital and Region Stockholm, St. Erik Eye Hospital and Karolinska University Hospital Stockholm and Region Västerbotten, Umeå University Hospital and Region Västmanlands Västerås Hospital, and Region Värmland, Centralsjukhuset in Karlstad and Region Gävleborg, Gävle Hospital and Region Örebro län, University Hospital Örebro and Region Östergötland, University Hospital, LinköpingRegion Dalarna, Falun Sjukhus

Following hospitals will work as “Satellite Hospital” to the University Hospital: Region Västra Götaland (Borås Hospital), Region Västerbotten (Östersund Hospital, Sunderbyn Hospital, Sundsvall Hospital), Region Uppsala (Eskilstuna Hopsital), Linköping Hospital (Norrköping Hospital).

3.1 Treatment Groups/Exposure

There will be two treatment arms in this study:

- Dexamethasone

Signed document (nngzKR)

- Placebo

3.2 Flow Chart

DROPROP STUDY Schedule									
	Treatment Phase (Main) Study							Extension Study	
**Procedures**	**ROP screening**	**Clinical visit**	**ROP Screening/ treatment**			**Clinical visit (40w PMA)**	**Termination. 2 weeks after last studydrops Visit[b]**	**Follow-up**	
	**Visit R0**	**Visit C1**	**Visit R1**	**Visit R2**	**Visit RX[a]**	**Visit C2**	**Visit T1**	**2 yrs Visit F1**	**5.5 yrs Visit F2**
Study Procedures and Data Collection									
Informed consent	X								
Eligibility	X								
Medical history/ demographics infant/mother	X								
ROP evaluations	X		X	X	X				
RetCam photography	X		X	X	X				
Randomization		X							
Dexamethasone/Placebo treatment dispense		X	X	X	X	X	X		
ROP treatment with laser and/or anti-VEGF			X[c]	X[c]	X[c]				
Retinal morphology with OCT								X	X
Intraocular pressure	X[d]		X[d]	X[d]			X[d]		
Visual outcome and refractive errors								X	X
MRI[e] + Peapod						X[e]			
Physical examination		X				X		X	X
Weight, Length, Head		X	X	X	X	X		X	X
Vital signs: SPO2, HR, BP		X					X		
Blood samples (optional)		X[f]	X[f]	X[f]	X[f]	X[f]	X[f]		
Saliva cortisol		X	X	X	X		X	X	X
Urine sample		X				X	X		
Adverse events	X	X	X	X	X	X	X		
Diagnoses and complications								X	X

Signed document (nngzKR)

Study termination		X	X	X	X	X	X	X

Visit R0= Visit before start of treatment
Visit C1 = Clinical visit 1 (start of treatment)
Visit R1 = 1 week after start of treatment
Visit R2 = 2 weeks after start of treatment
[a]Visit RX = ROP Screening and treatment as many as required in order to be correctly followed-up regarding the monitoring and treatment of the ROP disease, procedures performed weekly.
Visit C2 = Clinical visit 2 (40 weeks PMA)
Visit T1 = 2 weeks after end of treatment
[b] Main study termination visit occurs two weeks after the last treatment or at earliest at 40 weeks PMA when maximum ROP should have occurred.
[c] ROP treatment provided when required according to guidelines.
[d] Intraocular pressure will be measured before studydrops , 1 and 2 weeks after start of studydrops and 2 weeks after end of studydrops .
[e] if performed in clinical routine, PeaPod in Lund and Gothenburg
[f] if clinical sample is taken left over blood is collected for analyses

4 Study Populations

4.1 Intention-to-Treat Population

The intention-to-treat (ITT) population will include all randomized infants correctly included in the study and will be analysed on as-randomized basis. Infants wrongly included in the study will be excluded from the ITT population, but will be part of the safety population.

4.2 Per Protocol Population

The per protocol (PP) population will include all infants that are part of the ITT population that do not have any significant protocol violations. Protocol violations will be continuously collected in the study. The reasons for exclusion from the PP population will be reviewed and finalized during the clean file meeting before the database lock in a blinded manner. Per protocol analysis will be performed on as-treated basis.

4.3 Safety Population

All infants included in the study that have received at least one dose of either of the two study arms will be part of the safety population. Safety analysis will be performed on as-treated basis.

5 Study Variables

5.1 Baseline Characteristics Variables

Following infant baseline characteristics variables will be described:

- Gestational age (GA) at birth (weeks and days, weeks as unit)

Signed document (nngzKR)

- Sex (boy, girl)
- Birth weight (g)
- Birth length (cm)
- Birth head circumference (cm)
- Maximum eye pressure of right and left (mmHg)
- SPO2 (%)
- Heart rate (bpm)
- Age (postnatal and postmenstrual) when included in the study (week+days)
- ROP stage, zone, extent (different combinations) when included in the study
- Single/Twin/Triple

Following mother characteristics variables will be described:

- Mother's age (years)
- Any chronic disease (yes, no)
- Mother's diagnoses
    - Hypertension
    - Diabetes
    - Etc depending on the reported diagnoses
- Parity (1, 2, 3, …)
- In vitro fertilization (IVF) (yes, no)

5.2 Medical History

Following medical history will be collected prior to start:

- Pulmonary hemorrhage
- Pneumothorax
- Bronchopulmonary dysplasia
- Pulmonary hypertension
- Hypotension
- Circulatory instability
- Surgical patent ductus arteriosus (PDA)
- Medical PDA
- Hypoglycemia
- Hyperglycemia, insulin treated (>10 mmol/L at 2 independent timepoints)
- Necrotizing enterocolitis (NEC)
- Perforated gut
- Ileus
- Enterostomy
- Sepsis
- Meningitis
- Intraventricular hemorrhage IVH (1-3)
- Hydrocephalus

Signed document (nngzKR)

5.3 Pregnancy Data, Complications and Medications

Following data linked to pregnancy including complications and medications will be described:

- Preeclampsia (yes, no)
- Hemolysis, elevated liver enzymes, and low platelet count (HELLP) (yes, no)
- Gestational diabetes (yes, no)
- Preterm premature rupture of membranes (PPROM)
- Antenatal steroid treatment
- Chorioamnionitis
- Type of delivery (vaginal, cesarean section)

5.4 Postnatal Medications

Following postnatal medications will be recorded and described:

- Total dose of other steroids given (mg) – the harmonization of medication doses will be performed.

5.5 Efficacy/Outcome Variables

5.5.1 Primary Variable

The primary variable is the the proportion of infants with ROP progression to Type 1 ROP needing conventional treatment in each study arm.

5.5.2 Secondary Variables

The secondary variables in this study are:

The secondary objectives are:

- Time from randomization to Type 1 ROP or end of treatment phase study (all infants)
- Time from randomization to Type 1 ROP (infants without regression)
- The proportion of infants with any recurrences after laser/Anti-VEGF treatment (all infants)
- The number of recurrences per infant per follow-up time after laser/Anti-VEGF treatment (all infants)
- The proportion of infants with any recurrences after laser/Anti-VEGF treatment (infants progressed to Type 1 ROP)
- The number of recurrences per infant per follow-up time after laser/Anti-VEGF treatment (infants progressed to Type 1 ROP)
- Optic disc, cup and rim area at 40 weeks PMA and 2 and 5.5 years of age
- Retinal nerve fiber thickness at 40 weeks PMA and 2 and 5.5 years of age
- Macular thickness at 40 weeks PMA and 2 and 5.5 years of age
- Change in intraocular pressure (IOP) (mmHg) from before treatment, 1-2 weeks after treatment, and after end of treatment
- Visual acuity at 2 (Fix and follow) and 5.5 years (KM or HVOT) of age
- Refraction at 2 and 5.5 years of age
- Orthoptic status including motility, stereovision, presence of nystagmus and strabismus at 2 and 5.5 years of age

Signed document (nngzKR)

5.5.3 Exploratory Variables

Leftovers of blood from clinical blood sampling with regard to selected biomarkers and their relation to ROP development and treatment effect will be studied exploratively.

5.6 Safety Variables

5.6.1 Exposure and Compliance

Exposure of treatment will be described as number of weeks and number drops that were given to the infant. The provided number of drops will be described as a compliance measure given as a continuous variable and categorical variable <80%, 80-100%, >100%.

5.6.2 Adverse Events

Treatment emergent adverse events (AE) are those that have started on or after the first dose of Dexamethasone/Placebo in the study. The selected AEs will be collected continuously during the study and will be summarized by treatment group.

Following AEs will be collected:

- Pulmonary hemorrhage
- Pneumothorax
- Bronchopulmonary dysplasia
- Pulmonary hypertension
- Hypotension
- Circulatory instability
- Surgical PDA
- Medical PDA
- Hypoglycemia
- Hyperglycemia, insulin treated (>10 mmol/L at 2 independent timepoints)
- NEC
- Perforated gut
- Ileus
- Enterostomy
- Sepsis
- Meningitis
- IVH (1-3)
- Hydrocephalus
- Eye pressure >20 mmHg
- Abnormal growth >1SD (weight, height, or head circumference)

5.6.3 Laboratory Data

Following laboratory parameters will be collected:

- Urine glucose before start of study drops and two weeks after stop of study drops

Signed document (nngzKR)

5.6.4 Retinal Examination

ROP outcome will be collected from SWEDROP in addition to ROP status at start of study and after stop of study drops.

5.6.5 Intraocular Pressure

Intraocular pressure will be collected prior start of study drops, 1 and 2 weeks after start of drops and 2 weeks after stop of study drops and described as any pressure above 20 mmHg as well as longitudinal description.

5.6.6 Brain Magnetic Resonance Imaging

Brain magnetic resonance imaging (MRI) if performed in clinic will be analysed with regard to IVH and PVL.

5.6.7 Body Composition

Following body composition parameters will be collected weekly:

- Weight (g)
- Height (cm)
- Head circumference (cm)
- Peapod (Gothenburg) at 40 weeks PMA

5.6.8 Vital Signs

Following vital signs parameters will be collected:

- SPO2 (%)
- Heart rate (bpm)

6 Statistical Methods

6.1 Sample Size

Natural history of ROP has shown that 50% of the infants with stage 2, zone II ROP with hemorrhage or stage 3, zone II develop Type 1 ROP needing treatment (1) . It is anticipated that the number of infants needing treatment with laser and/or Anti-VEGF will be reduced by 60% in this study. Assuming 50% infants needing ROP treatment in the Placebo arm and 20% in the Dexamethasone treated arm, alpha 0.05, using two-sided Fisher's exact test, would require 45 infants to be included per study arm (1:1 ratio), in total 90 infants excluding drop-outs, in order to achieve a power of 80%. Taking into account 10% drop-outs, 50 infants per study arm, 100 infants in total will be needed to be included.

6.2 General Methodology

All analyses will be performed using either SAS software version 9.4 or later (SAS Institute Inc, Cary, NC, USA) or SPSS software version 26.0 or later (IBM Corp, Armonk, NY) or R (R Core Team, 2022 or later).

For descriptive purposes mean, standard deviation (SD), median, minimum and maximum will be presented for continuous variables and number and percentages for categorical variables.

For test between the two study arms regarding infant's characteristics at study start, Fisher's exact test will be used for dichotomous variables, Mantel-Haenszel Chi-square trend test for ordered categorical variables, Chi-

Signed document (nngzKR)

square test for non-ordered categorical variables and Fisher's non-parametric permutation test for continuous variables.

All efficacy evaluations will be adjusted for center (used as strata in randomization) and gestational age (the most prominent risk factor).

Evaluation of difference between the two study arms with respect to binary outcomes will be performed using logistic regression, and graphically presented by bar charts. Missing data for early discontinuations will be considered as no event. Sensitivity analysis will study the missing values considered being event instead.

Evaluation of time to event data between the two study arms will be performed using Cox proportional hazards model, and graphically presented by Kaplan-Meier graphs. Missing data will be handled through censoring.

Evaluation of number of recurrences per follow-up time between the two study arms will be described by event rates (number of events divided by the number of follow-up days) and will be compared using Poisson regression.

Evaluation of difference between the two study arms with respect to continuous outcomes will be performed using analysis of covariance (ANCOVA) if analyses assumptions might be shown fulfilled, otherwise, unadjusted analyses will be performed using Fisher's non-parametric permutation test, and graphically presented by scatter plots or boxplots.

Evaluation of difference between the two study arms with respect to continuous outcomes repeatedly measured over time will be performed using mixed models for repeated measures (MMRM) if analyses assumptions might be shown fulfilled, adjusted for before treatment value (beside center and GA), otherwise, unadjusted analyses will be performed using Fisher's non-parametric permutation test, and graphically presented by scatter plots or boxplots.

All efficacy variables will be evaluated on the ITT and PP populations, ITT being the main analysis. All safety variables will be evaluated on the safety population.

All tests will be two-tailed.

The primary analysis will be considered confirmed if $p<0.05$. All other analyses will be exploratory.

6.3 Adjustment for Type I Error

There will be only confirmatory analysis in this trial, i.e. the primary analysis, where alpha of 0.05 will be applied. All other analyses will be considered exploratory.

6.4 Handling of Missing Data

Missing data for the event outcomes is not expected. In case of missing data for these variables, missing event will be considered as no event. A sensitivity analysis will be performed handling missing data as event. The continuous variables will be handled by using mixed models for repeated measures that is able to handle the missing data as if it was present completely at random.

Signed document (nngzKR)

6.5 Confounders/Covariates

Covariates in the analyses will be the randomization strata, i.e. the study center, and the most prominent risk factor for the outcome, gestational age.

7 Statistical Analyses

7.1 Patient Disposition and Datasets Analysed

The number of randomized infants and those included in different study populations will be described by the treatment group, including reason for early discontinuation from the study.

7.2 Protocol Deviations

The list of major protocol deviations will be provided by treatment group leading to exclusion from the PP population.

7.3 Baseline Characteristics

Baseline characteristics will be described by the treatment group and tested according to the methods described in the general methodology above.

7.4 Medical History

Medical history will be collected for selected comorbidities and will be described by the treatment group.

7.5 Pregnancy Data, Complications and Medications

Pregnancy data, complications and medications will be described by the treatment group and tested according to the methods described in the general methodology above.

7.6 Postnatal Medications

The use of postnatal steroids will be described by treatment group.

7.7 Efficacy Analyses

7.7.1 Primary Efficacy Analysis

The primary outcome, the proportion of infants with ROP progression to Type 1 ROP needing conventional treatment, will be evaluated for difference between the two treatment groups using logistic regression, adjusted for the center used as stratifying variable in the randomization procedure and GA that is the most prominent predictor for Type 1 ROP. In this analysis, missing data for early discontinuations will be handled as *non-events*. Odds-ratio (OR) with 95% confidence intervals (CIs) will be presented along with its p-value. If

Signed document (nngzKR)

p<0.05 for the analysis performed on ITT population on as-randomized basis, the primary analysis will be considered confirmed.

Analysis on PP population on as treated basis will be performed as complementary analysis.

Analysis on ITT population imputing *event* to missing data will be performed as sensitivity analysis.

Graphically, the proportion of infants with Type 1 ROP in the two treatment groups will be described by bar-charts.

Descriptive data will be provided separately per each center, and the interaction between center and treatment arm will be studied. Smaller centers including <10 infants will be grouped together. Any center differences will be reported and discussed.

7.7.2 Secondary Efficacy Analyses

The secondary efficacy analyses performed on the secondary outcomes will be evaluated according to the methods described in the general methodology above. These analyses will not be included in the confirmatory tests but described with appropriate estimates and 95% CI. Analyses adjusted for center (smaller centers including <10 infants will be grouped together) and GA will be made.

The main analysis will be performed on the ITT population on as-randomized basis.

The complementary analysis will be performed on the PP population on as-treated basis.

7.7.3 Exploratory Efficacy Analyses

Leftovers of blood from clinical blood sampling with regard to selected biomarkers and their relation to ROP development and treatment effect will be studied exploratively using MMRM.

7.7.4 Subgroup Analyses

Following subgroup analyses will be performed on the primary variable:

- Center (smaller centers including <10 infants will be grouped together)
- ROP severity at baseline (stage 2 zone 2, or stage 3)
- Postnatal age (PNA) at baseline
- PMA at baseline
- Number of drops per day
- GA categories (<24 weeks, 25-26 weeks, ≥27 weeks)
- Birth weight categories (below and above median)
- Parenteral nutrition (<14 days, ≥14 days)
- Relation to DIGIROP risk estimates

7.8 Safety Analyses

7.8.1 Exposure and Compliance

Exposure and compliance will be described by treatment group.

7.8.2 Adverse Events

Only treatment-emergent AEs will be included in the summaries for the safety population. A summary of infants with reported at least one of the following AEs will be presented in an overview table:

Signed document (nngzKR)

- Any AE
- Any SAE
- Any treatment-related AE
- Any treatment-related SAE
- Any AE leading to discontinuation
- Death

Summaries per selected AE diagnoses presenting n (%) of AEs and n (%) of infants with at least one AE will be provided for:

- All AEs (includes all serious and non-serious AEs)
- All AEs by maximum reported intensity
- All AEs by causality
- All SAEs
- All AEs leading to discontinuation
- All AEs leading to death

7.8.3 Laboratory Data

Laboratory data will be summaries over time by treatment group.

7.8.4 Retinal Examination

Postnatal and postmenstrual age at resolved ROP will be presented by treatment group.

7.8.5 Intraocular Pressure

Intraocular pressure will be summaries over time by treatment group.

7.8.6 Brain Magnetic Resonance Imaging

Outcome of focal brain lesions and other remarks will be described by treatment group.

7.8.7 Body Composition

Body composition will be summaries over time by treatment group.

7.8.8 Vital Signs

Vital signs will be summaries over time by treatment group.

8 Interim Analyses

A DMC will be appointed in this study including an independent group of experts in the area that are not the Study Sponsor or part of the study's Steering Committee; Gunnar Jakobsson MD PhD (voting), ophthalmologist, Boubou Hallberg MD PhD, (voting), neonatologist, and Prof Max Petzold PhD (voting), statistician, will scrutinize the data according to below prepared by a non-voting statistical programmer.

Two planned closed DMC meetings will be held, the first one when 35% of the infants have been followed up until the main study end, and the second one when 70% of the infants have been followed up until the main study end. At both time points the DMC will have the possibility to analyse data for efficacy (benefit) and for safety (harm). Ó'Brian-Flemming group sequential boundaries on positive side will be applied for evaluation of

Signed document (nngzKR)

potential stopping for benefit, and z-value exceeding 2.40 will be applied for evaluation of potential stopping for harm. DMC may decide given the descriptive data to not perform formal statistical interim tests if there is no sign of need for formal analyses for stopping for harm and stopping for efficacy. The DMC will not have in its responsibility to stop the study for futility. Given that the formal statistical interim tests are performed, the final p-values must be updated, based on the number of interim tests performed. DMC may also, if found necessary, schedule additional DMC meetings in-between planned closed meetings.

Information about study results will be strictly held within the closed DMC group. After a closed DMC meeting, the Sponsor and the study's Steering Committee will only receive a recommendation to *Stop* or *Continue* the study. The decision on any actualization will be made by the study's Steering Committee.

A detailed plan describing the DMC's work will be available in a DMC charter, written and signed off before the start of the first DMC analyses.

9 Changes from Study Protocol

Subgroup analyses are added compared to the protocol version 4.

10 Planned Tables and Figures

Table Number	Table Title
**Table 1.1**	Patient disposition and data sets analyzed (ITT population)
**Table 1.2**	Protocol violation leading to exclusion from PP population (ITT population)
**Table 2.1**	Baseline characteristics (ITT population)
**Table 2.2**	Baseline characteristics (PP population)
**Table 3.1**	Medical history (ITT population)
**Table 3.2**	Pregnancy data, complications, and medications (ITT population)
**Table 3.3**	Postnatal medications (ITT population)
**Table 4.1**	Primary efficacy analysis (ITT population)
**Table 4.2**	Analyses of the primary efficacy variable (PP population)
**Table 5.1**	Secondary efficacy analyses (ITT population)
**Table 5.2**	Secondary efficacy analyses (PP population)
**Table 6.1**	Exploratory analyses (ITT population)
**Table 6.2**	Exploratory analyses (PP population)
**Table 7**	Subgroup analyses of the primary variable (ITT population)
**Table 8**	Exposure and compliance (Safety population)
**Table 9.1**	Summary of adverse events (Safety population)
**Table 9.2**	Adverse events by diagnosis (Safety population)
**Table 9.3**	Adverse events by diagnosis and maximum severity (Safety population)
**Table 9.4**	Adverse events by diagnosis and causality assessment (Safety population)
**Table 9.5**	Serious adverse events by diagnosis (Safety population)
**Table 9.6**	Adverse events leading to discontinuation (Safety population)
**Table 10**	Laboratory data (Safety population)
**Table 11**	Retinal examination (Safety population)
**Table 12**	Intraocular pressure (Safety population)

Signed document (nngzKR)

**Table 13**	Brain MRI (Safety population)
**Table 14**	Body composition (Safety population)
**Table 15**	Vital signs (Safety population)

**Figure Number**	**Figure Title**
**Figure 1.1**	Primary analysis (ITT population)
**Figure 1.2**	Primary analysis (PP population)
**Figure x.1**	Secondary analysis of xxx (ITT population)
**Figure x.2**	Secondary analysis of xxx (PP population)
**Figure x**	Incidence of adverse events by treatment group (Safety population)

Signed document (nngzKR)

Verification appendix

Digital signing service provided by https://www.resly.se. The original document, raw signatures and the event logs are attached as hidden attachments to this document. Open this document in Adobe Reader to verify the signature. The certificate used to sign this document is issued by Entrust for Resly AB and is listed in the European Union Trusted Lists (EUTL). Printed version of this document is not legally valid.

ID: 65ef6ae8b2c4719f0194764e **Finalized at:** 2024-03-11 21:46:43 CET

Title: SAP DROPROP v1.0.pdf

Digest: nngzKRF6f1TuZhydUayuKdU6coey2hVZFfwJRmETKSA=

Initiated by: aldina.pivodic@apnc.se (*aldina.pivodic@apnc.se*) via APNC Sweden AB 559351-3186

Signees:

- Ann Hellström signed at 2024-03-11 21:46:42 CET with Swedish BankID (**19590609-XXXX**)
- Aldina Pivodic signed at 2024-03-11 21:36:57 CET with Swedish BankID (**19780221-XXXX**)
