# Supplementary material for: Dexamethasone Eye Drops to Prevent Treatment-Requiring Retinopathy of Prematurity: The DROPROP Randomized Clinical Trial
Source: JAMA Pediatr. 2026 Aug 3:e263247. Online ahead of print. doi: 10.1001/jamapediatrics.2026.3247 (PMC13434969; doi:10.1001/jamapediatrics.2026.3247)
Supplement: Supplementary file 3 [file jamapediatr-e263247-s003.pdf]

Supplemental Online Content

Hellström A, Petrishka-Lozenska M, Sjöbom U, et al; DROPROP Collaboration Group. Dexamethasone eye drops to prevent treatment-requiring retinopathy of prematurity: the DROPROP randomized clinical trial. *JAMA Pediatr*. Published online August 3, 2026. doi:10.1001/jamapediatrics.2026.3247

eAppendix 1. Research Nurses Involved in the DROPROP Study

eAppendix 2. DROPROP Dose-Adjusting Scheme

eFigure 1. Box Plot of IOP Over Time (Safety Population)

eFigure 2. Box Plots of Weight (g) and SDS Over Time (Safety Population)

eFigure 3. Box Plots of Height (cm) and SDS Over Time (Safety Population)

eFigure 4. Box Plots of Head Circumference (cm) and SDS Over Time (Safety Population)

eFigure 5. Box Plot of Salivary Cortisol Concentration (ITT Population)

eTable 1. Infants' Medical History (ITT Population)

eTable 2. Mothers' Pregnancy Data and Complications (ITT Population)

eTable 3. Baseline Characteristics (PP Population)

eTable 4. Baseline Characteristics (Safety Population)

eTable 5. Exposure (Safety Population)

eTable 6. Robustness Analyses of the Primary Outcome (PP Population)

eTable 7. Subgroup Analysis of the Primary Outcome (ITT Population)

eTable 8. Analyses of Secondary Outcomes (PP Population)

This supplemental material has been provided by the authors to give readers additional information about their work.

© 2026 Hellström A et al. *JAMA Pediatrics.*

eAppendix 1. Research Nurses Involved in the DROPROP Study

Carola Pfeiffer Mosesson[1], Anne Rosenkvist[1], Anna-Lena Fransson[1], Christina Ragnarsson[1], Ann-Charlotte Andersson[1], Camilla Halzius[2], Therese Kjellin[2], Veronica Norlin[3], Anna Stål[3], Gabriella Lindén[4], Elenore Åström[4], Emma Karlsson[4], Anette Blomstedt[5], Freja Norrblom[5], Monica Firus[5], Anette Larsson[6], Liselotte Timlöv[6], Isabel Neyra Marklund[7], Gunilla Strinnholm[7], Marie Lindvall[7], Ella Törnroth[8], Elin Morin[8], Frida Vallius[9], Malin Nordin[9], Ulrica Gustafsson[10], Gunilla Varildengen[11], Martina Carlsen Misic[12]

[1] Section for Ophthalmology, Department of Clinical Neuroscience, Institute of Neuroscience and Physiology, Sahlgrenska Academy, University of Gothenburg, Gothenburg, Sweden

[2] Department of Neonatology, Karolinska Institute and University Hospital, Stockholm, Sweden

[3] Department of Ophthalmology, Skaraborg Hospital, Skövde, Sweden.

[4] Department of Pediatrics, Region Jönköping County, Jönköping, Sweden.

[5] Department of Women's and Children's Health, Uppsala University, Uppsala, Sweden.

[6] Department of Pediatrics, Region Sörmland, Eskilstuna, Sweden.

[7] Department of Clinical Sciences, Pediatrics, Umeå University, Umeå, Sweden.

[8] Department of Pediatrics, Region Östergötland, Linköping, Sweden.

[9] Department of Pediatrics, Region Gävleborg, Gävle, Sweden.

[10] Department of Pediatrics, Region Värmland, Karlstad, Sweden.

[11] Department of Pediatrics, Region Västra Götaland, Borås, Sweden.

[12] Department of Pediatrics, Region Örebro län, Örebro, Sweden.

© 2026 Hellström A et al. *JAMA Pediatrics.*

eAppendix 2. DROPROP Dose-Adjusting Scheme

**Administration schedule for eye drops according to ROP severity.**	
	**Number of drops per day**
ROP stage 1-2 without plus disease in zone I	1 drop every other day
ROP stage 2 without plus disease in posterior Zone II	1 drop every other day
ROP stage 3 without plus disease in zone II, with or without notch in zone 1	1 drop daily
Type 1 ROP* fulfilling treatment criteria	If anti-VEGF injection is planned, eye drops should be immediately withdrawn.
Treatment and post treatment	Routines at the clinic
Abbreviations: ROP; retinopathy of prematurity, VEGF; vascular endothelial growth factor. *Any stage of ROP in zone I, with plus disease; ROP stage 3 in zone I, without plus disease or ROP stage 2 or 3 in zone II, with plus disease.	

© 2026 Hellström A et al. *JAMA Pediatrics.*

eFigure 1. Box Plot of IOP Over Time (Safety Population)

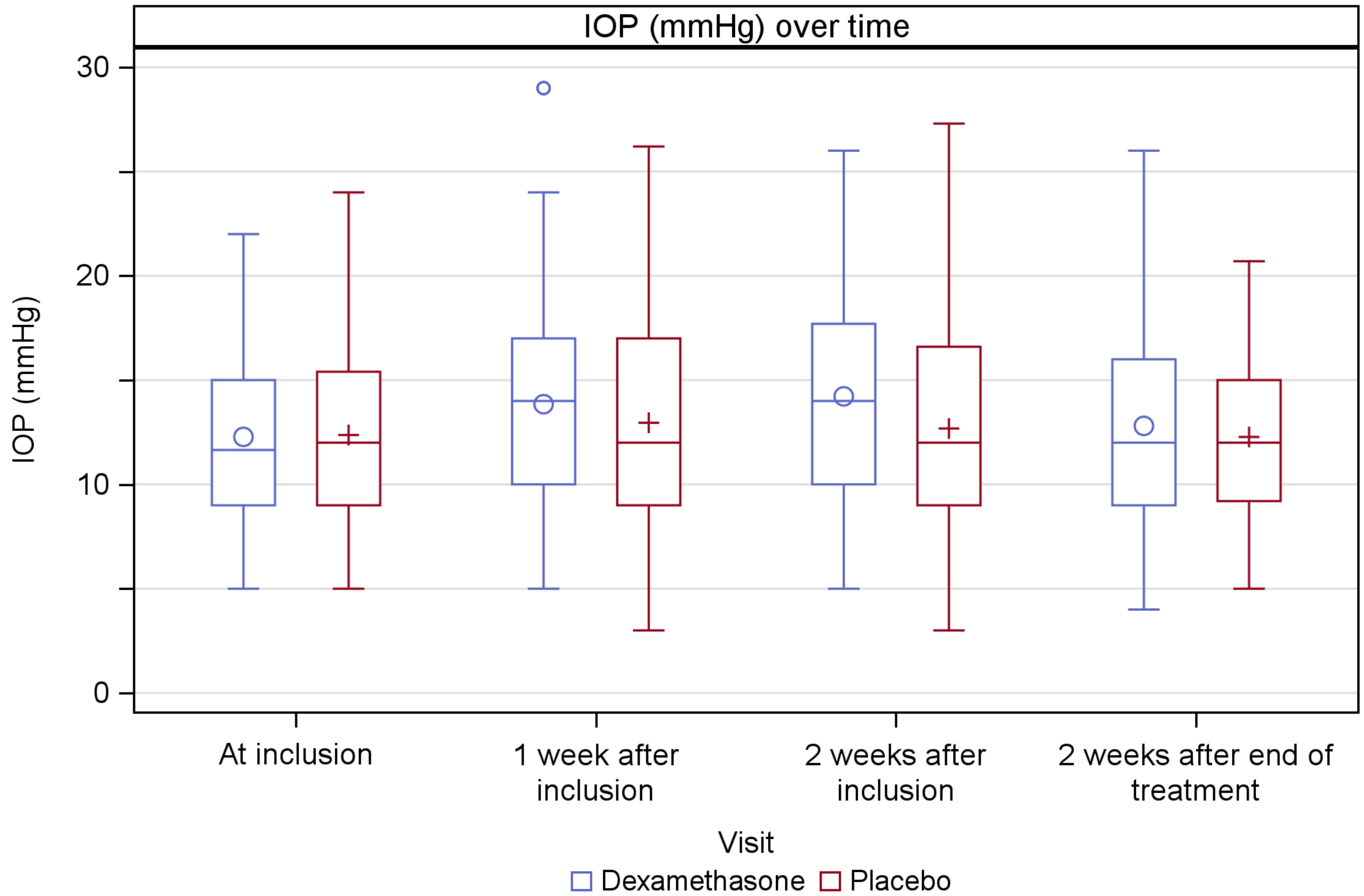

© 2026 Hellström A et al. *JAMA Pediatrics.*

eFigure 2. Box Plots of Weight (g) and SDS Over Time (Safety Population)

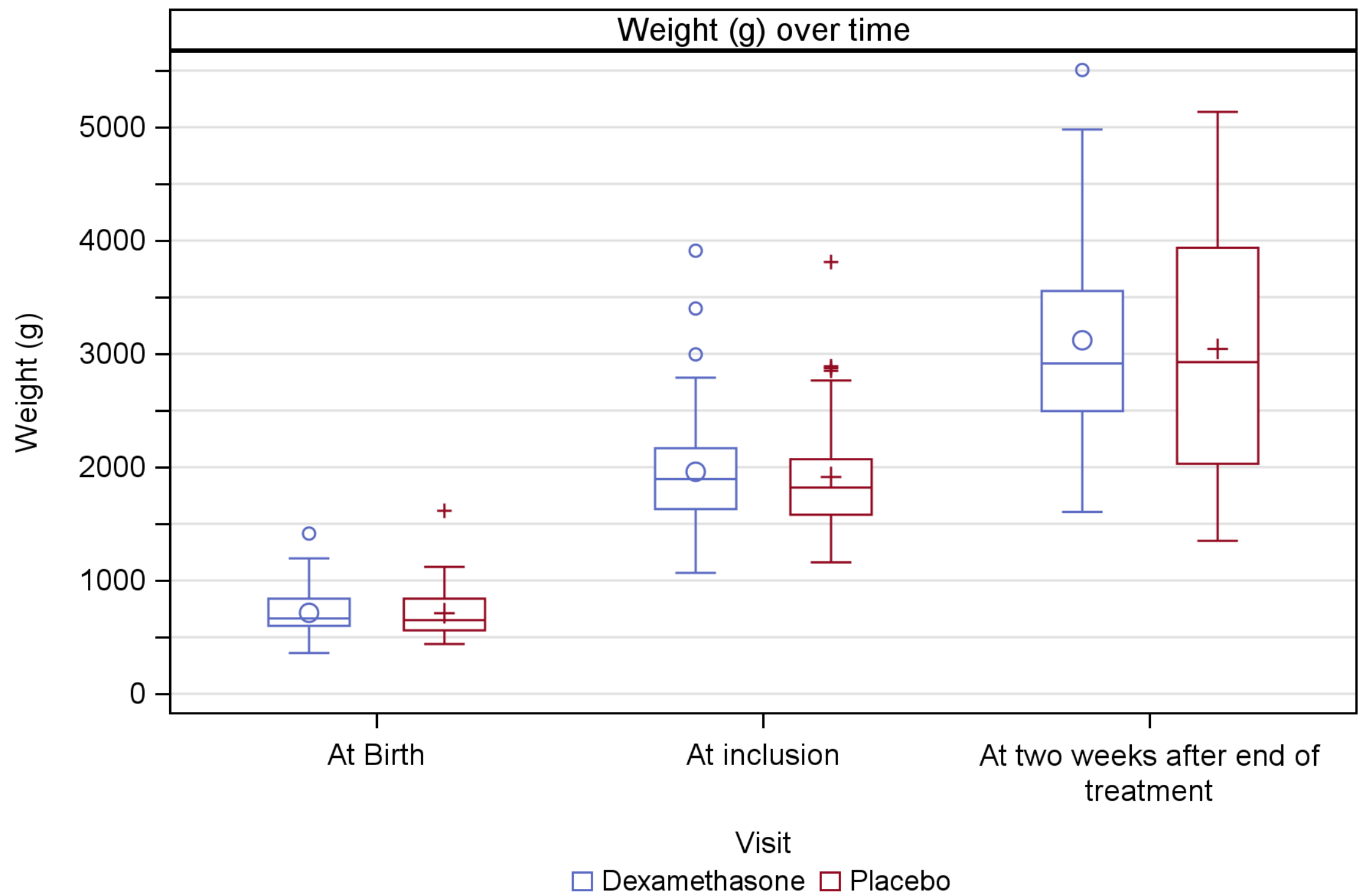

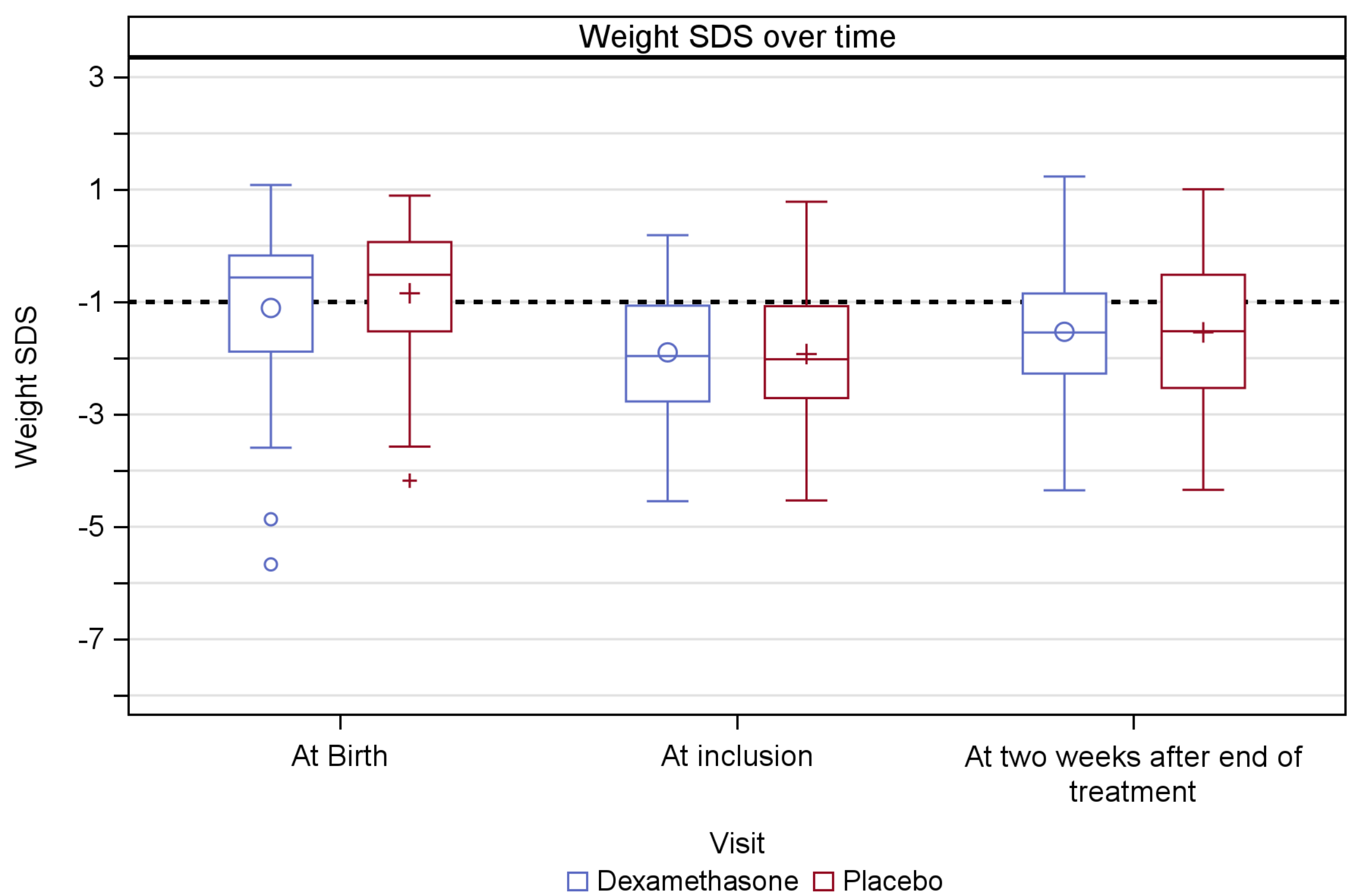

© 2026 Hellström A et al. *JAMA Pediatrics.*

eFigure 3. Box Plots of Height (cm) and SDS Over Time (Safety Population)

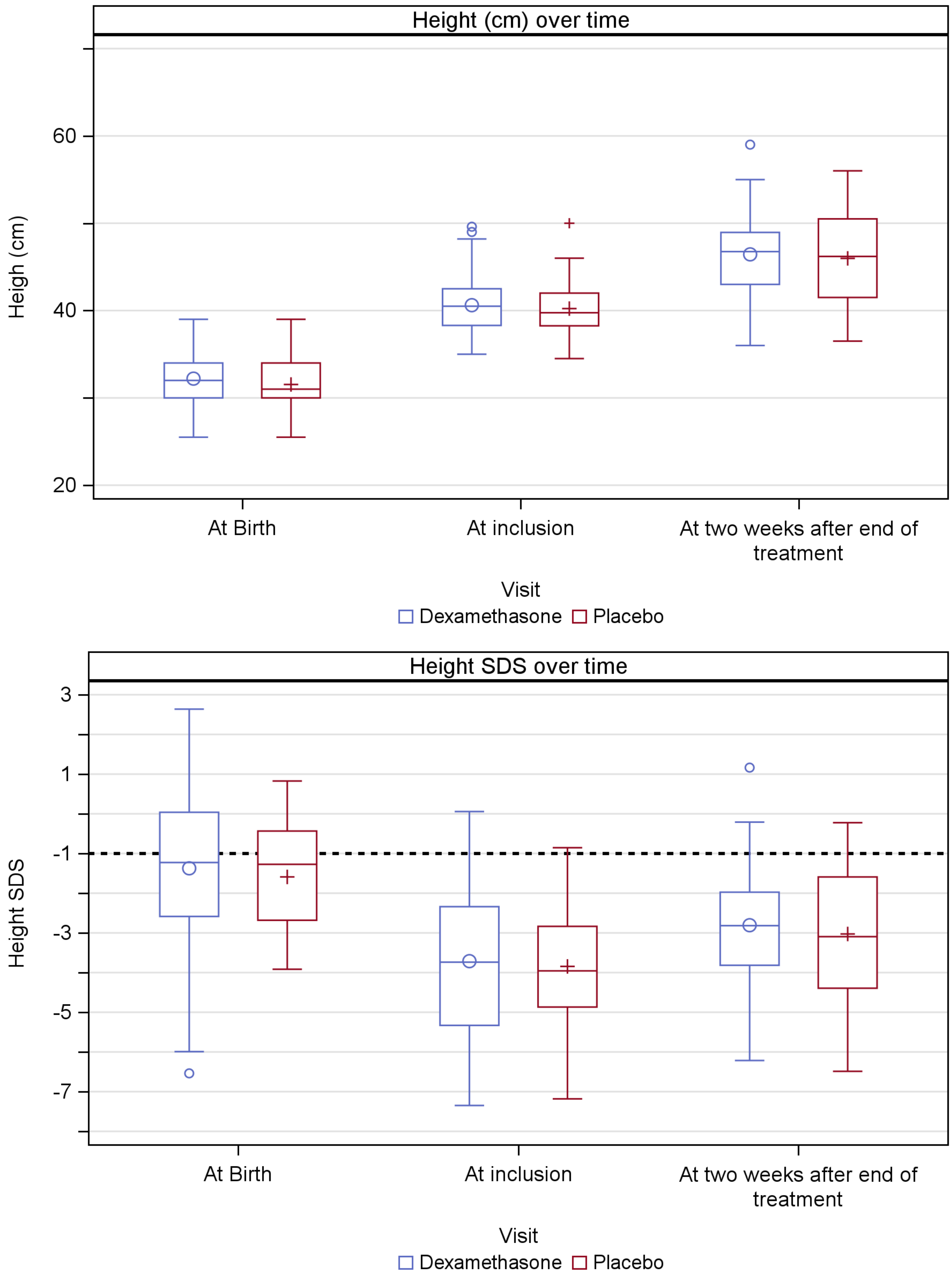

© 2026 Hellström A et al. *JAMA Pediatrics.*

eFigure 4. Box Plots of Head Circumference (cm) and SDS Over Time (Safety Population)

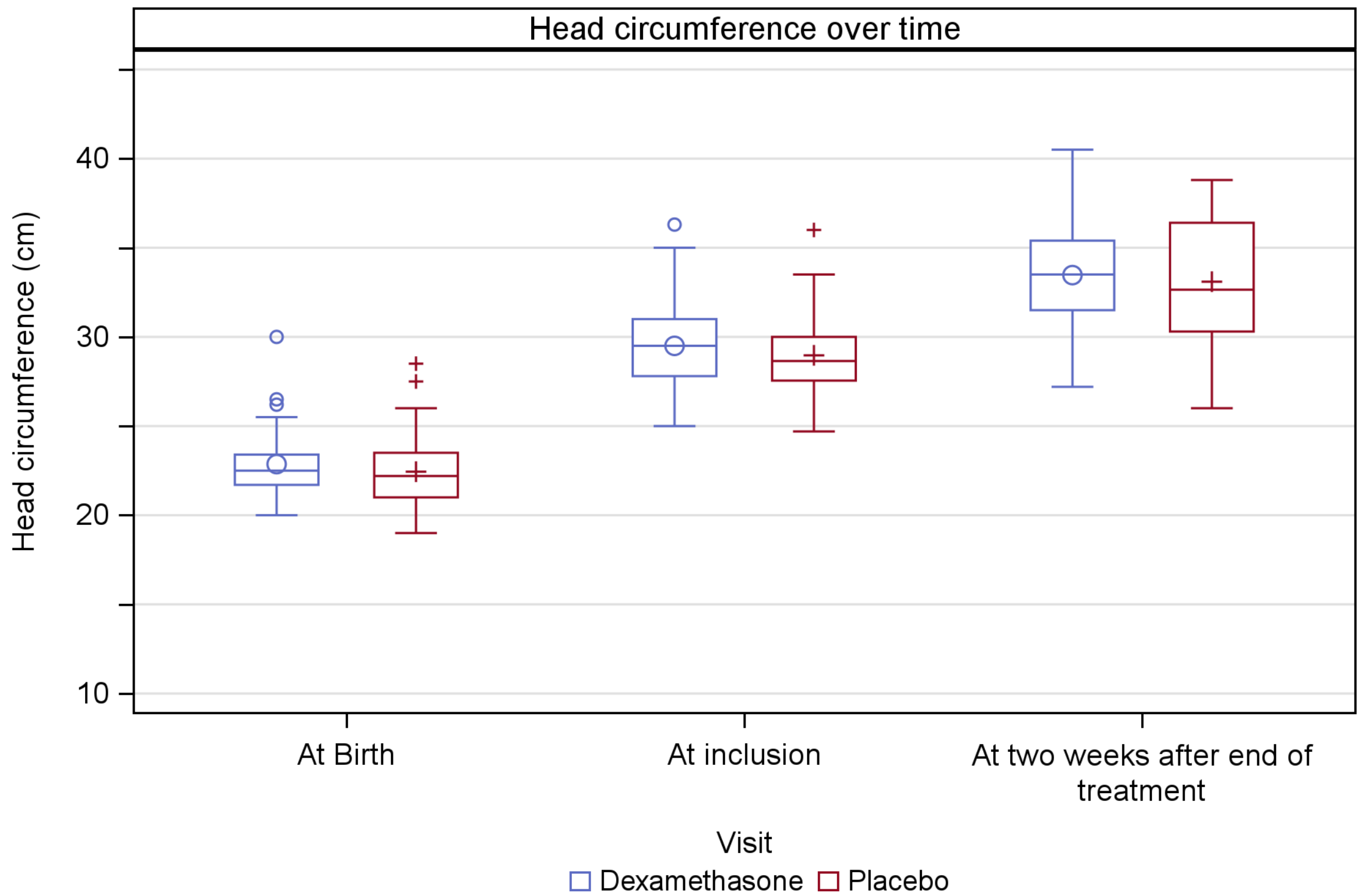

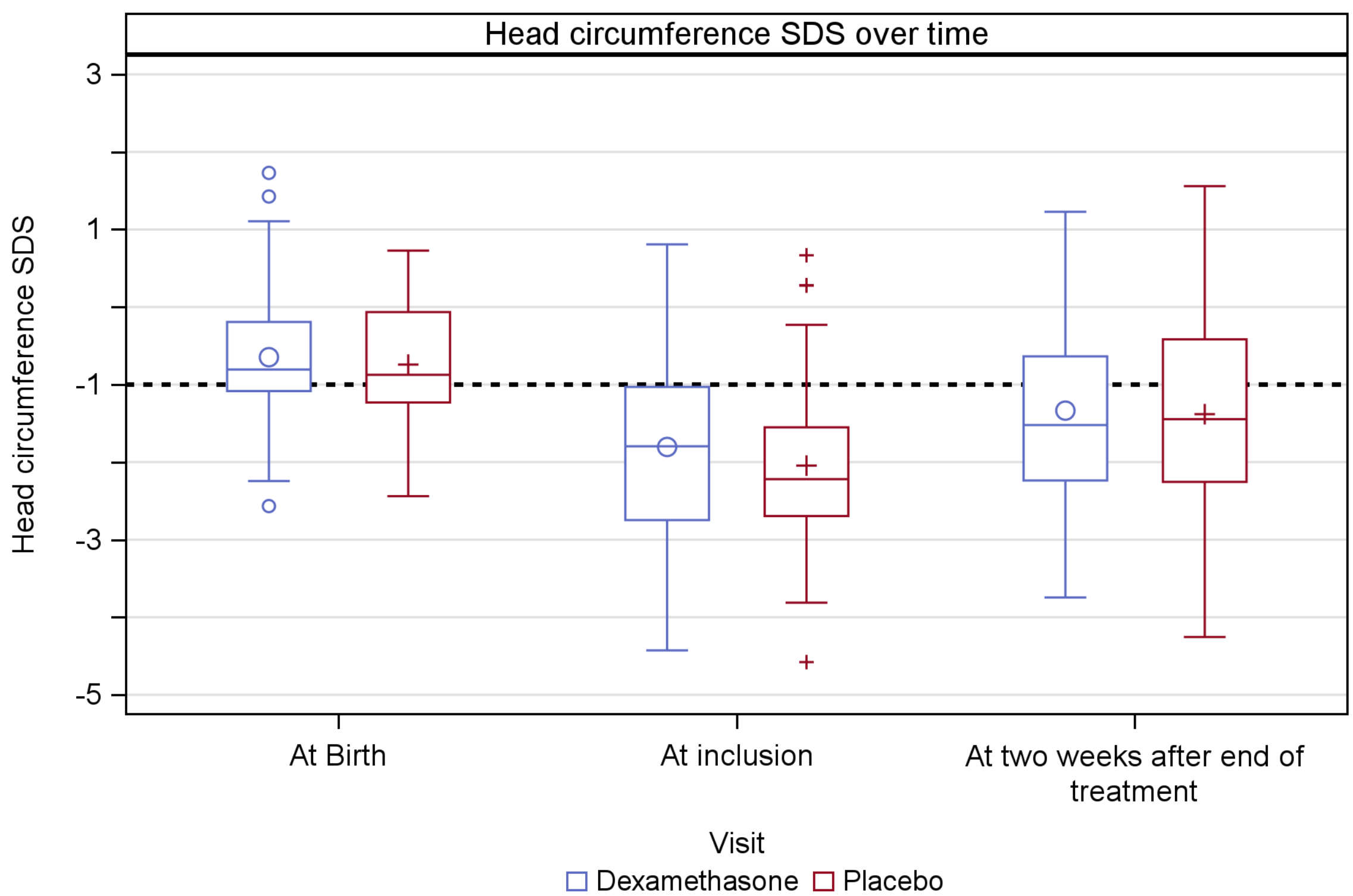

© 2026 Hellström A et al. *JAMA Pediatrics.*

eFigure 5. Box Plot of Salivary Cortisol Concentration (ITT Population)

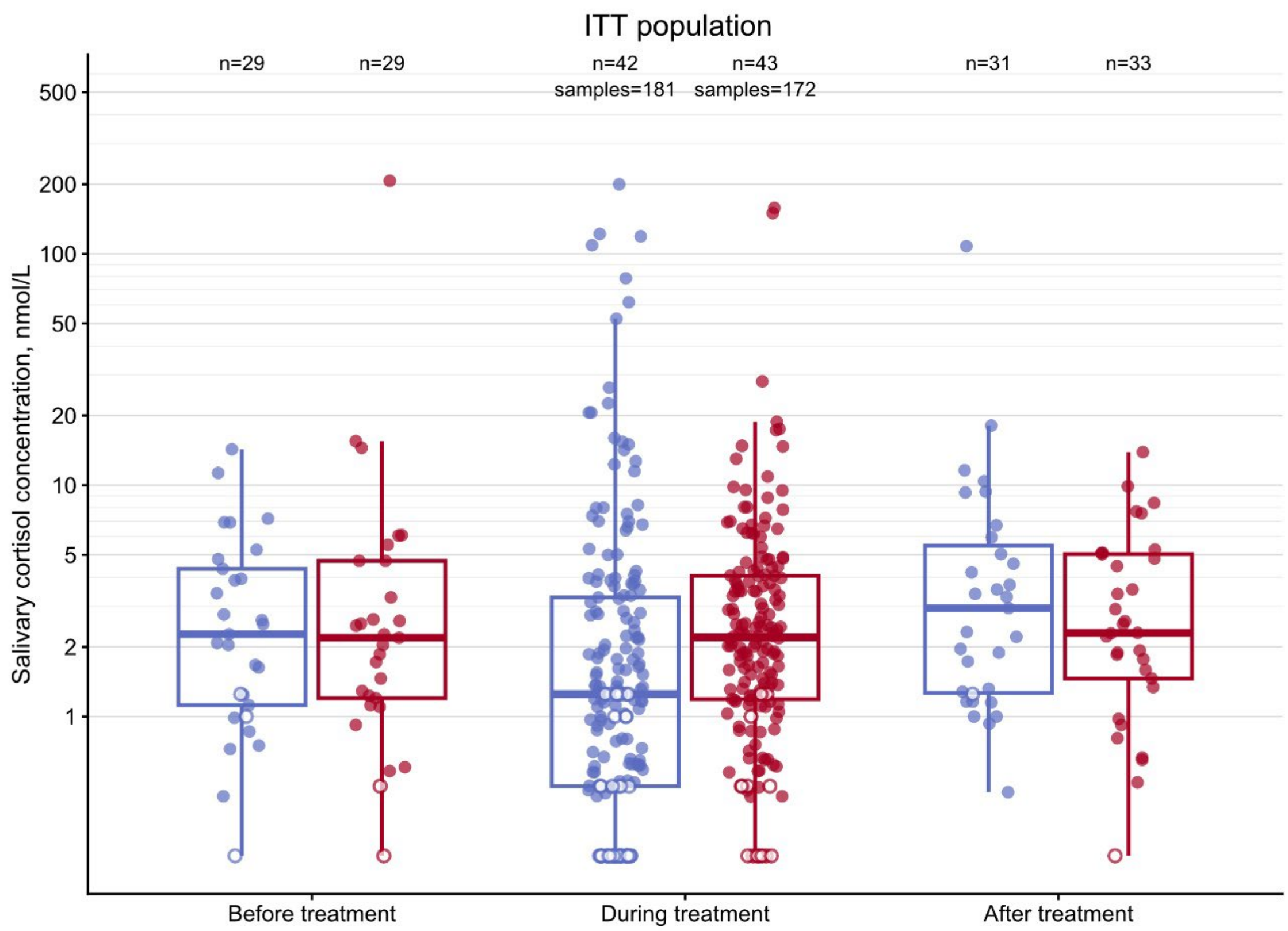

© 2026 Hellström A et al. *JAMA Pediatrics.*

eTable 1. Infants' Medical History (ITT Population)

Variable	Dexamethasone N=50	Placebo N=50
Lungbleed	3 (6.0%)	2 (4.0%)
Pneumothorax	0 (0.0%)	1 (2.0%)
Pulmonary hemorrhage	5 (10.0%)	5 (10.0%)
Hypotension	9 (18.0%)	9 (18.0%)
Circulatory instability	0 (0.0%)	3 (6.0%)
Surgical patent ductus arteriosus (PDA)	34 (68.0%)	31 (62.0%)
Medical PDA	2 (4.0%)	2 (4.0%)
Hypoglycemia	9 (18.0%)	12 (24.0%)
Hyperglycemia, insulin treated (>10 mmol/L at 2 independent timepoints)	29 (58.0%)	28 (56.0%)
Necrotizing enterocolitis (NEC)	6 (12.0%)	8 (16.0%)
Perforated gut	6 (12.0%)	4 (8.0%)
Ileus	3 (6.0%)	4 (8.0%)
Enterostomy	4 (8.0%)	8 (16.0%)
Sepsis	24 (49.0%)	25 (51.0%)
Meningitis	0 (0.0%)	1 (2.0%)
Intraventricular hemorrhage IVH (1-3)	19 (38.0%)	22 (44.0%)
Hydrocephalus	3 (6.0%)	4 (8.0%)
Data are presented as mean, standard deviation (SD), median, minimum, maximum and number of observations for continuous variables and number and percentages for categorical variables.		

eTable 2. Mothers' Pregnancy Data and Complications (ITT Population)

Variable	Dexamethasone N=50	Placebo N=50
Preeclampsia	8 (16.0%)	8 (16.7%)
Hemolysis, elevated liver enzymes, and low platelet count (HELLP)	1 (2.0%)	2 (4.2%)
Gestational diabetes	3 (6.0%)	4 (8.3%)
Preterm premature rupture of membranes (PPROM)	13 (26.0%)	21 (45.7%)
Antenatal steroid treatment	48 (96.0%)	42 (87.5%)
Chorioamnionitis	11 (22.0%)	8 (17.0%)
Vaginal delivery	21 (42.0%)	18 (36.7%)
Cesarean section	29 (58.0%)	30 (61.2%)
Data are presented as mean, standard deviation (SD), median, minimum, maximum and number of observations for continuous variables and number and percentages for categorical variables.		

© 2026 Hellström A et al. *JAMA Pediatrics.*

eTable 3. Baseline Characteristics (PP Population)

	Dexamethasone<br>N=48	Placebo<br>N=49
Infants' characteristics		
Gestational age at birth (weeks)	25.3±1.4<br>25.1 (22.9 - 29.1)<br>n=48	24.9±1.5<br>24.7 (22.6 - 29.9)<br>n=49
Gestational age at birth (cat.)		
<25 weeks	22 (45.8%)	26 (53.1%)
25-26 weeks	19 (39.6%)	21 (42.9%)
≥27 weeks	7 (14.6%)	2 (4.1%)
Sex		
Boy	30 (62.5%)	27 (55.1%)
Girl	18 (37.5%)	22 (44.9%)
Twin	9 (18.8%)	10 (20.4%)
ROP stage at inclusion		
Stage 2 posterior zone II	12 (25.0%)	11 (22.4%)
Stage 3	36 (75.0%)	38 (77.6%)
Parenteral nutrition (cat.)		
<14 days	16 (33.3%)	17 (36.2%)
≥14 days	32 (66.7%)	30 (63.8%)
Missing	0	2
Site		
Göteborg	29 (60.4%)	23 (46.9%)
Jönköping	3 (6.3%)	6 (12.2%)
Linköping	1 (2.1%)	2 (4.1%)
Stockholm	8 (16.7%)	8 (16.3%)
Uppsala	5 (10.4%)	8 (16.3%)
Umeå	2 (4.2%)	2 (4.1%)
Weight at birth (g)	725.5±195.7<br>677.0 (360.0 - 1414.0)<br>n=48	711.0±211.0<br>650.0 (439.0 - 1615.0)<br>n=49
Weight at birth (cat.)		
≤660 g	22 (45.8%)	26 (53.1%)
>660 g	26 (54.2%)	23 (46.9%)
Height at birth (cm)	32.4±2.8<br>33.0 (25.5 - 39.0)<br>n=46	31.5±2.9<br>31.0 (25.5 - 39.0)<br>n=47
Head circumference at birth (cm)	23.0±1.8<br>22.5 (20.5 - 30.0)<br>n=46	22.4±2.0<br>22.2 (19.0 - 28.5)<br>n=47
Postnatal age at inclusion (weeks)	9.2±2.0<br>8.6 (6.1 - 15.0)<br>n=48	9.2±1.5<br>9.3 (6.3 - 13.1)<br>n=49
Postnatal age at inclusion (cat.)		
≤9 weeks	27 (56.3%)	23 (46.9%)
>9 weeks	21 (43.8%)	26 (53.1%)

© 2026 Hellström A et al. *JAMA Pediatrics.*

	Dexamethasone N=48	Placebo N=49
Postmenstrual age at inclusion (weeks)	34.5±2.2<br>34.3 (31.3 - 41.3)<br>n=48	34.2±1.7<br>33.9 (30.9 - 39.7)<br>n=49
Postmenstrual age at inclusion (cat.)		
≤34 weeks	21 (43.8%)	28 (57.1%)
>34 weeks	27 (56.3%)	21 (42.9%)
Maximum eye pressure of right eye (mmHg)	11.9±3.6<br>12.0 (5.0 - 19.1)<br>n=46	12.1±4.5<br>12.0 (5.0 - 24.0)<br>n=43
Maximum eye pressure of left eye (mmHg)	12.7±4.2<br>11.0 (5.0 - 22.0)<br>n=46	12.7±4.3<br>12.5 (5.0 - 22.0)<br>n=43
SPO2 (%)	94.1±3.1<br>94.0 (88.0 - 100.0)<br>n=48	93.1±5.0<br>94.0 (65.0 - 100.0)<br>n=48
Heart rate (bpm)	150.0±11.6<br>150.0 (117.0 - 170.0)<br>n=48	153.2±11.3<br>153.0 (126.0 - 180.0)<br>n=47
Mothers' characteristics		
Mother's age	33.0±5.1<br>34.0 (22.0 - 42.0)<br>n=48	31.8±4.8<br>31.0 (21.0 - 42.0)<br>n=49
Any chronic disease	13 (27.1%)	12 (24.5%)
Parity (1, 2, 3, …)	1.6±1.1<br>1.0 (1.0 - 6.0)<br>n=48	1.6±0.9<br>1.0 (0.0 - 6.0)<br>n=47
In vitro fertilization (IVF)	7 (14.6%)	13 (29.5%)
Data are presented as mean, standard deviation ± SD, median, minimum, and maximum for continuous variables and number and percentages for categorical variables.		

© 2026 Hellström A et al. *JAMA Pediatrics.*

eTable 4. Baseline Characteristics (Safety Population)

	Dexamethasone<br>N=51	Placebo<br>N=49
Infants' characteristics		
Gestational age at birth (weeks)	25.2±1.4<br>25.0 (22.9 - 29.1)<br>n=51	24.9±1.5<br>24.7 (22.6 - 29.9)<br>n=49
Gestational age at birth (cat.)		
<25 weeks	25 (49.0%)	26 (53.1%)
25-26 weeks	19 (37.3%)	21 (42.9%)
≥27 weeks	7 (13.7%)	2 (4.1%)
Sex		
Boy	31 (60.8%)	27 (55.1%)
Girl	20 (39.2%)	22 (44.9%)
Twin	9 (17.6%)	10 (20.4%)
ROP stage at inclusion		
Stage 2 posterior zone II	14 (27.5%)	11 (22.4%)
Stage 3	37 (72.5%)	38 (77.6%)
Parenteral nutrition (cat.)		
<14 days	17 (33.3%)	17 (36.2%)
≥14 days	34 (66.7%)	30 (63.8%)
Missing	0	2
Site		
Göteborg	29 (56.9%)	23 (46.9%)
Jönköping	3 (5.9%)	6 (12.2%)
Linköping	1 (2.0%)	2 (4.1%)
Stockholm	11 (21.6%)	8 (16.3%)
Uppsala	5 (9.8%)	8 (16.3%)
Umeå	2 (3.9%)	2 (4.1%)
Weight at birth (g)	714.7±195.3<br>665.0 (360.0 - 1414.0)<br>n=51	711.0±211.0<br>650.0 (439.0 - 1615.0)<br>n=49
Weight at birth (cat.)		
≤660 g	25 (49.0%)	26 (53.1%)
>660 g	26 (51.0%)	23 (46.9%)
Height at birth (cm)	32.2±2.9<br>32.0 (25.5 - 39.0)<br>n=49	31.5±2.9<br>31.0 (25.5 - 39.0)<br>n=47
Head circumference at birth (cm)	22.9±1.8<br>22.5 (20.0 - 30.0)<br>n=49	22.4±2.0<br>22.2 (19.0 - 28.5)<br>n=47
Postnatal age at inclusion (weeks)	9.3±2.0<br>8.7 (6.1 - 15.0)<br>n=51	9.2±1.5<br>9.3 (6.3 - 13.1)<br>n=49
Postnatal age at inclusion (cat.)		
≤9 weeks	28 (54.9%)	23 (46.9%)

© 2026 Hellström A et al. *JAMA Pediatrics.*

	Dexamethasone<br>N=51	Placebo<br>N=49
>9 weeks	23 (45.1%)	26 (53.1%)
Postmenstrual age at inclusion (weeks)	34.5±2.2<br>34.3 (31.3 - 41.3)<br>n=51	34.2±1.7<br>33.9 (30.9 - 39.7)<br>n=49
Postmenstrual age at inclusion (cat.)		
≤34 weeks	23 (45.1%)	28 (57.1%)
>34 weeks	28 (54.9%)	21 (42.9%)
Maximum eye pressure of right eye (mmHg)	11.9±3.5<br>12.0 (5.0 - 19.1)<br>n=49	12.1±4.5<br>12.0 (5.0 - 24.0)<br>n=43
Maximum eye pressure of left eye (mmHg)	12.7±4.1<br>11.0 (5.0 - 22.0)<br>n=49	12.7±4.3<br>12.5 (5.0 - 22.0)<br>n=43
SPO2 (%)	94.1±3.1<br>94.0 (88.0 - 100.0)<br>n=50	93.1±5.0<br>94.0 (65.0 - 100.0)<br>n=48
Heart rate (bpm)	150.0±11.4<br>150.0 (117.0 - 170.0)<br>n=50	153.2±11.3<br>153.0 (126.0 - 180.0)<br>n=47
Mothers' characteristics		
Mother's age	32.9±5.0<br>34.0 (22.0 - 42.0)<br>n=51	31.8±4.8<br>31.0 (21.0 - 42.0)<br>n=49
Any chronic disease	13 (25.5%)	12 (24.5%)
Parity (1, 2, 3, …)	1.6±1.1<br>1.0 (1.0 - 6.0)<br>n=51	1.6±0.9<br>1.0 (0.0 - 6.0)<br>n=47
In vitro fertilization (IVF)	8 (15.7%)	13 (29.5%)
Data are presented as mean, standard deviation ± SD, median, minimum, and maximum for continuous variables and number and percentages for categorical variables.		

© 2026 Hellström A et al. *JAMA Pediatrics.*

eTable 5. Exposure (Safety Population)

	Dexamethasone<br>N=51	Placebo<br>N=49
Number of drops	70.5±48.6<br>64.0 (4.0 - 168.0)<br>n=51	74.2±47.5<br>66.0 (10.0 - 166.0)<br>n=49
Number of drops (cat.)		
≤64 drops	26 (51.0%)	24 (49.0%)
>64 drops	25 (49.0%)	25 (51.0%)
Number of days on drops	43.5±26.7<br>37.0 (3.0 - 89.0)<br>n=51	41.7±26.6<br>36.0 (5.0 - 88.0)<br>n=49
Number of days on drops (cat.)		
≤35 days	24 (47.1%)	23 (46.9%)
>35 days	27 (52.9%)	26 (53.1%)
Drops per day	1.6±0.4<br>1.7 (0.5 - 2.0)<br>n=51	1.8±0.2<br>1.9 (1.0 - 2.0)<br>n=49
Drops per day (cat.)		
≤1.5 drops/day	21 (41.2%)	5 (10.2%)
>1.5 - <2 drops/day	22 (43.1%)	28 (57.1%)
≥2 drops/day	8 (15.7%)	16 (32.7%)

Data are presented as mean, standard deviation ± SD, median, minimum, and maximum for continuous variables and number and percentages for categorical variables.

eTable 6. Robustness Analyses of the Primary Outcome (PP Population)

	Dexamethasone		Placebo		Unadjusted analyses	Adjusted analyses*
	N	n (%) events	N	n (%) events	OR (95% CI)	OR (95% CI)
PP population						
Robustness analysis						
Incidence of Type 1 ROP	48	9 (18.8%)	49	19 (38.8%)	0.36 (0.14-0.92)	0.40 (0.15-1.05)
Post-hoc sensitivity analysis						
Incidence of ROP treatment	48	13 (27.1%)	49	21 (42.9%)	0.50 (0.21-1.16)	0.53 (0.22-1.27)
Incidence of Type 1 ROP (excluding infants treated without Type 1 ROP)	44	9 (20.5%)	47	19 (40.4%)	0.38 (0.15-0.97)	0.43 (0.16-1.14)

* Primary analysis is the analysis of type 1 ROP adjusted for gestational age (fixed) and site (random) using logistic regression, for the ITT population.

© 2026 Hellström A et al. *JAMA Pediatrics.*

eTable 7. Subgroup Analysis of the Primary Outcome (ITT Population)

	Dexamethasone		Placebo		Logistic regression *
**Subgroup**	**N**	**n (%) events**	**N**	**n (%) events**	**OR (95% CI)**
Site					
Göteborg	29	6 (20.7%)	23	10 (43.5%)	0.36 (0.11-1.19)
Stockholm	10	3 (30.0%)	9	3 (33.3%)	0.87 (0.13-5.85)
Uppsala	5	0 (0.0%)	8	2 (25.0%)	0.24 (0.01-8.05)
Others	6	1 (16.7%)	10	4 (40.0%)	0.39 (0.04-4.02)
ROP severity at inclusion					
Stage 2 posterior zone II	13	3 (23.1%)	12	4 (33.3%)	0.63 (0.11-3.55)
Stage 3	37	7 (18.9%)	38	15 (39.5%)	0.37 (0.13-1.05)
Postnatal age at inclusion					
≤9 weeks	28	6 (21.4%)	23	7 (30.4%)	0.64 (0.18-2.22)
>9 weeks	22	4 (18.2%)	27	12 (44.4%)	0.30 (0.08-1.10)
Postmenstrual age at inclusion					
≤34 weeks	22	6 (27.3%)	29	13 (44.8%)	0.48 (0.15-1.57)
>34 weeks	28	4 (14.3%)	21	6 (28.6%)	0.44 (0.11-1.75)
Gestational age at birth					
<25 weeks	24	6 (25.0%)	27	14 (51.9%)	0.33 (0.10-1.07)
25-26 weeks	19	4 (21.1%)	21	4 (19.0%)	1.13 (0.25-5.12)
≥27 weeks	7	0 (0.0%)	2	1 (50.0%)	0.07 (0.00-4.14)
Birth weight					
≤660 g	24	7 (29.2%)	27	10 (37.0%)	0.71 (0.22-2.30)
>660 g	26	3 (11.5%)	23	9 (39.1%)	0.23 (0.06-0.94)
Parenteral nutrition					
<14 days	17	0 (0.0%)	17	7 (41.2%)	0.04 (0.00-0.84)
≥14 days	33	10 (30.3%)	31	11 (35.5%)	0.80 (0.28-2.26)
Number of drops					
≤64 drops	25	8 (32.0%)	25	16 (64.0%)	0.28 (0.09-0.90)
>64 drops	25	2 (8.0%)	25	3 (12.0%)	0.68 (0.12-3.96)
Number of days on drops					
≤35 days	23	8 (34.8%)	24	16 (66.7%)	0.28 (0.08-0.94)
>35 days	27	2 (7.4%)	26	3 (11.5%)	0.66 (0.11-3.78)
Drops per day					
≤1.5 drops/day	20	2 (10.0%)	6	1 (16.7%)	0.50 (0.05-5.32)
>1.5 - <2 drops/day	21	6 (28.6%)	28	7 (25.0%)	1.20 (0.34-4.25)
≥2 drops/day	9	2 (22.2%)	16	11 (68.8%)	0.16 (0.03-1.00)

© 2026 Hellström A et al. *JAMA Pediatrics.*

eTable 8. Analyses of Secondary Outcomes (PP Population)

	Dexamethasone		Placebo		Unadjusted analyses	Adjusted analyses*
	N	n (%) events	N	n (%) events	Effect size (95% CI)	Effect size (95% CI)
Secondary analyses						
Time to Type 1 ROP[1]	48	9 (18.8%)	49	19 (38.8%)	0.46 (0.21-1.01)	0.54 (0.24-1.21)
Time to ROP treatment[1]	48	13 (27.1%)	49	21 (42.9%)	0.59 (0.30-1.18)	0.65 (0.32-1.32)
Proportion of infants with any recurrence after Laser (n=2) or Anti-VEGF (n=5)[2]	48	2 (4.2%)	49	5 (10.2%)	0.38 (0.07-2.08)	0.49 (0.08-2.85)

* Analysis adjusted for gestational age (fixed) and site (random), using Firth correction for small number of events.
[1] Time-to-event outcomes described by n (%) and analyses performed using Cox regression. Hazard ratio with 95% CI presented as effect size.
[2] Binary outcomes described by n (%) and analyses performed using logistic regression. Odds ratio with 95% CI presented as effect size.

© 2026 Hellström A et al. *JAMA Pediatrics.*
