# Supplementary material for: Dexamethasone Eye Drops to Prevent Treatment-Requiring Retinopathy of Prematurity: The DROPROP Randomized Clinical Trial
Source: JAMA Pediatr. 2026 Aug 3:e263247. Online ahead of print. doi: 10.1001/jamapediatrics.2026.3247 (PMC13434969; doi:10.1001/jamapediatrics.2026.3247)
Supplement: Supplementary file 4 [file jamapediatr-e263247-s004.pdf]

Supplemental Online Content: Nonauthor Collaborators
*First name, last name, and suffix (if applicable) are required and will appear in PubMed.

*Group Name(s): DROPROP Collaboration Group							
***First Name and Middle Initial(s)**	***Last Name**	***Suffix (eg, Jr, III)**	Academic Degrees	Institution	Location (city, state/province, country)	Role or Contribution, eg, chair, principal investigator	Group (if more than 1 Group listed in the byline) and/or Subgroup (eg, Steering Committee)
Liv	Vallin		MD	Department of Pediatrics, Institute of Clinical Sciences, Sahlgrenska Academy, University of Gothenburg and Department of Neonatology, Region Västra Götaland, The Queen Silvia Children's Hospital, Sahlgrenska University Hospital	Gothenburg, Region Västra Götaland, Sweden	Investigator	
Ingrid	Hansen-Pupp		MD, PhD	Department of Clinical Sciences, Pediatrics, Lund University, Skåne University Hospital	Lund, South Region, Sweden	Chair	
Kristina	Teär Fahnehjelm		MD, PhD	Department of Clinical Neuroscience, Karolinska Institutet and Department of Paediatric Ophthalmology, Strabismus and Electrophysiology, St. Erik Eye Hospital	Stockholm, Region Stockholm, Sweden	Investigator	
Eva	Albinsson		MD	Department of Women's and Children's Health, Uppsala University	Karlstad, Region Varmland, Sweden	Investigator	
Erik	Normann		MD, PhD	Department of Women's and Children's Health, Uppsala University	Uppsala, Region Uppsala, Sweden	Co-PI	
Afsaneh	Alibakhshi		MD	Department of Neonatology, Karolinska University Hospital,	Borås, Region Västra Götaland, Sweden	Investigator	

Supplemental Online Content: Nonauthor Collaborators
*First name, last name, and suffix (if applicable) are required and will appear in PubMed.

***First Name and Middle Initial(s)**	***Last Name**	***Suffix (eg, Jr, III)**	Academic Degrees	Institution	Location (city, state/province, country)	Role or Contribution, eg, chair, principal investigator	Group (if more than 1 Group listed in the byline) and/or Subgroup (eg, Steering Committee)
Nikica	Tomašić		MD	Department of Neonatology, Karolinska University Hospital and Division of Pediatric Endocrinology and Metabolism, Karolinska University Hospital	Stockholm, Region Stockholm, Sweden	Investigator	
Fredrik	Ingemansson		MD, PhD	Department of Pediatrics, Länssjukhuset Ryhov	Jönköping, Region Jönköping County, Sweden	Investigator	
Pierfrancesco	Mirabelli		MD, PhD	Department of Ophthalmology and Department of Biomedical and Clinical Sciences, Linköping University	Linköping, Region Östergötland, Sweden	PI	
Per	Odelberg		MD, PhD	Department of Pediatrics, Linköping University	Linköping, Region Östergötland, Sweden	Investigator	
Despina	Tsamadou		MD	Department of Clinical Neuroscience, Karolinska Institute and Department of Ophthalmology, Örebro University Hospital	Sockholm, Region Stockholm and Örebro, Region Örebro County, Sweden	Investigator	
Evangelos	Tsigkoulis		MD	Department of Ophthalmology and Department of Biomedical and Clinical Sciences, Linköping University	Linköping, Region Östergötland, Sweden	Investigator	
Johanna	Kopparström		MD	Ophthalmology Department at Uddevalla Hospital	Uddevalla, Region Västra Götaland, Sweden	Investigator	
Sofie	Eriksson		MD	Department of Ophthalmology, Central Hospital Karlstad	Karlstad, Region Varmland, Sweden	Co-PI	
Karin	Hochard Sandgren		MD	Ophthalmology, Department of Clinical Sciences, Umeå University	Umeå, Region Västerbotten, Sweden	Co-PI	

Supplemental Online Content: Nonauthor Collaborators
*First name, last name, and suffix (if applicable) are required and will appear in PubMed.

***First Name and Middle Initial(s)**	***Last Name**	***Suffix (eg, Jr, III)**	Academic Degrees	Institution	Location (city, state/province, country)	Role or Contribution, eg, chair, principal investigator	Group (if more than 1 Group listed in the byline) and/or Subgroup (eg, Steering Committee)
Aikaterni	Massou		MD	Department of Ophthalmology, Gävle Hospital	Gävle, Region Gävleborg, Sweden	Investigator	
Ylva	Friberg-Riad		MD	Department of Ophthalmology, Skaraborg Hospital	Skövde, Region Västra Götaland, Sweden	PI	
Athanasia	Skriapa-Manta		MD, PhD	Department of Clinical Neuroscience, Karolinska Institutet and Department of Paediatric Ophthalmology, Strabismus and Electrophysiology, St. Erik Eye Hospital	Stockholm, Region Stockholm, Sweden	Investigator	
Liv	Lind		MD	Ophthalmology Clinic, Östersund Hospital	Östersund, Region Jämtland Härjedalen, Sweden	Investigator	
Ulrika	Lidén		MD	Department of Ophthalmology, Region Sörmland	Eskilstuna, Sweden	Investigator	
Linnéa	Holmberg		MSc	APNC Sweden, Mölndal	Region Västra Götaland, Sweden	Statistician	
