# Supplementary material for: Dexamethasone Eye Drops to Prevent Treatment-Requiring Retinopathy of Prematurity: The DROPROP Randomized Clinical Trial
Source: JAMA Pediatr. 2026 Aug 3:e263247. Online ahead of print. doi: 10.1001/jamapediatrics.2026.3247 (PMC13434969; doi:10.1001/jamapediatrics.2026.3247)
Supplement: Supplementary file 5 [file jamapediatr-e263247-s005.pdf]

Data Sharing Statement

Hellström. Dexamethasone Eye Drops to Prevent Treatment-Requiring Retinopathy of Prematurity. *JAMA Pediatr*. Published August 03, 2026. doi:10.1001/jamapediatrics.2026.3247

Data
Additional Information: EudraCT#2020-004933-19, registered in January 2021, and CTIS #2023-505318-97-00, registered in August 2023.
Data available: No
